# Randomised controlled trial of simvastatin treatment for autism in young children with neurofibromatosis type 1 (SANTA)

**DOI:** 10.1186/s13229-018-0190-z

**Published:** 2018-02-22

**Authors:** Stavros Stivaros, Shruti Garg, Maria Tziraki, Ying Cai, Owen Thomas, Joseph Mellor, Andrew A. Morris, Carly Jim, Karolina Szumanska-Ryt, Laura M Parkes, Hamied A. Haroon, Daniela Montaldi, Nicholas Webb, John Keane, Francisco X. Castellanos, Alcino J. Silva, Sue Huson, Stephen Williams, D. Gareth Evans, Richard Emsley, Jonathan Green, SANTA Consortium, Suzanne Campbell, Ruth Ellicott, Emma Harrison, Akhtar Kapasi, Giangiacomo Mercatali, Rachel Moon, Hannah Tobin, Srilaxmi Velandy, Rose Wagstaffe, Emma Burkitt-Wright, Grace Vassallo, Siobhan West, Judith Eelloo, Eileen Hupton, Sonia Patel, Elizabeth Howard, Karen Tricker, Lauren Lewis, Angus Dobbie, Ruth Drimer, Saghira Malik Sharif, Zahabiyah Bassi, Jamuna Acharya, Wayne Lam, Neil Harrower, Oliver Quarrell, Alyson Bradbury, Miranda Splitt, Susan Musson, Rachel Jones, Helen Bethell, Catherine Prem, Karen Horridge, Christine Steiger

**Affiliations:** 1 Academic Unit of Paediatric Radiology, Royal Manchester Children’s Hospital, Central Manchester University Hospitals NHS Foundation Trust, Manchester Academic Health Sciences Centre, Manchester, UK; 2 Division of Informatics, Imaging and Data Sciences, School of Health Sciences, Faculty of Biology, Medicine and Health, University of Manchester, Manchester Academic Health Science Centre, Manchester, UK; 3 Division of Neuroscience and Experimental Psychology, School of Biological Sciences, Faculty of Biology, Medicine and Health, University of Manchester, Manchester Academic Health Science Centre, Manchester University NHS Foundation Trust, Greater Manchester Mental Health NHS Foundation Trust, Room 3.311, Jean McFarlane Building, Oxford Road, Manchester, M13 9PL UK; 4 Division of Neuroscience and Experimental Psychology, School of Biological Sciences, Faculty of Biology, Medicine and Health, University of Manchester, Manchester Academic Health Science Centre, Manchester, UK; 5 Departments of Neurobiology, Psychiatry and Biobehavioral Sciences and Psychology, Integrative Center for Learning and Memory, Brain Research Institute, Brain Research Institute, University of California, California, LA 90095 USA; 6 Academic Unit of Radiology, Salford Royal Foundation NHS Trust, Manchester Academic Health Sciences Centre, Manchester, UK; 7 Computer Science, University of Manchester, Manchester, UK; 8 Manchester University NHS Foundation Trust, Manchester Academic Health Sciences Centre, Manchester, UK; 9 Manchester Metropolitan University, Manchester, UK; 10 Department of Paediatric Nephrology, Royal Manchester Children’s Hospital, Manchester University NHS Foundation Trust, Academic Health Sciences Centre, Manchester, UK; 11 Hassenfeld Children’s Hospital at NYU Langone, Nathan S. Kline Institute for Psychiatric Research, New York, USA; 12 Manchester Centre for Genomic Medicine, St Mary’s Hospital, Manchester University NHS Foundation Trust, Academic Health Sciences Centre, Manchester, UK; 13 Centre for Biostatistics, School of Health Sciences, Faculty of Biology, Medicine and Health, University of Manchester, Manchester, UK

**Keywords:** Autism, Neurofibromatosis type 1, Neuroimaging, Randomised controlled trial, Statin, Simvastatin

## Abstract

**Background:**

Neurofibromatosis 1 (NF1) is a monogenic model for syndromic autism. Statins rescue the social and cognitive phenotype in animal knockout models, but translational trials with subjects > 8 years using cognition/behaviour outcomes have shown mixed results. This trial breaks new ground by studying statin effects for the first time in younger children with NF1 and co-morbid autism and by using multiparametric imaging outcomes.

**Methods:**

A single-site triple-blind RCT of simvastatin vs. placebo was done. Assessment (baseline and 12-week endpoint) included peripheral MAPK assay, awake magnetic resonance imaging spectroscopy (MRS; GABA and glutamate+glutamine (Glx)), arterial spin labelling (ASL), apparent diffusion coefficient (ADC), resting state functional MRI, and autism behavioural outcomes (Aberrant Behaviour Checklist and Clinical Global Impression).

**Results:**

Thirty subjects had a mean age of 8.1 years (SD 1.8). Simvastatin was well tolerated. The amount of imaging data varied by test. Simvastatin treatment was associated with (i) increased frontal white matter MRS GABA (*t*(12) = − 2.12, *p* = .055), GABA/Glx ratio (*t*(12) = − 2.78, *p* = .016), and reduced grey nuclei Glx (ANCOVA *p* < 0.05, Mann-Whitney *p* < 0.01); (ii) increased ASL perfusion in ventral diencephalon (Mann-Whitney *p* < 0.01); and (iii) decreased ADC in cingulate gyrus (Mann-Whitney *p* < 0.01). Machine-learning classification of imaging outcomes achieved 79% (*p* < .05) accuracy differentiating groups at endpoint against chance level (64%, *p* = 0.25) at baseline. Three of 12 (25%) simvastatin cases compared to none in placebo met ‘clinical responder’ criteria for behavioural outcome.

**Conclusions:**

We show feasibility of peripheral MAPK assay and autism symptom measurement, but the study was not powered to test effectiveness. Multiparametric imaging suggests possible simvastatin effects in brain areas previously associated with NF1 pathophysiology and the social brain network.

**Trial registration:**

EU Clinical Trial Register (EudraCT) 2012-005742-38 (www.clinicaltrialsregister.eu)

**Electronic supplementary material:**

The online version of this article (10.1186/s13229-018-0190-z) contains supplementary material, which is available to authorized users.

## Background

Neurofibromatosis 1 (NF1) is the most common autosomal dominant single-gene neurodevelopmental disorder with incidence of 1:2700, [1] caused by loss of function mutations in the NF1 gene on chromosome 17q11.2 encoding for neurofibromin. Although identified by neurocutaneous manifestations, morbidity in childhood NF1 usually relates to cognitive, social and behavioural difficulties, with moderate cognitive impairment and academic underachievement in about 80% [2] and attention-deficit/hyperactivity disorder (ADHD) in 38–50% [2, 3]. Recent evidence of autism spectrum disorder (ASD) prevalence of ~ 25% with partial traits in a further 20% [4, 5] support NF1 as a promising single-gene syndromic model for understanding ASD pathology [6].

The neurobiology of the social and learning deficits in NF1 has been studied in N*f*1^+/−^ mouse models and recent in-human studies [7]. Neurofibromin is a negative regulator of rat-sarcoma viral oncogene homologue (Ras); loss of neurofibromin causes disinhibition of the RasMAPK pathway with consequent GABA/glutamate disequilibrium, impairment in long-term potentiation (LTP) and synaptic plasticity [8]. Upregulation of the Ras pathway can also directly affect myelin formation and axonal integrity [9] and dysregulate nitric oxide signalling pathways in oligodendrocytes [10]. Recent diffusion tensor imaging (DTI) study in human NF1 [11] demonstrated increased apparent diffusion coefficient (ADC) values localised in the caudate and other deep grey nuclei, diencephalon and frontal white matter in NF1 children compared to controls, consistent with decreased neuronal density or myelin sheath disorganisation; the extent of these effects was associated with neurological symptoms. Other imaging studies in human NF1 have identified reduced cortical GABA [12, 13], reduced cerebral perfusion [14], alteration in diffusion-weighted imaging [15] and abnormal network connectivity on resting state fMRI [16, 17].

This emerging understanding of NF1 neural system pathophysiology from animal and human studies has provided a rationale for experimental intervention trials. Compensatory downregulation of Ras activation can be achieved by blocking its farnesylisation, using 3-hydroxy-3-methylglutaryl coenzyme A (HMG-CoA) reductase inhibitors (statins). Attenuation of Ras activity in *Nf1*^*+/−*^ mouse models using lovastatin [18] or, alternatively, through genetic co-deletion of the Pak1 gene (*Nf1*^*+/−*^*, Pak1*^*+/−*^) [7] rescues the biochemical, electrophysiological and behavioural deficits, including normalisation of social memory and autism-like behavioural phenotypes. Convergence in effect of these two methods supports the specificity of the target mechanism. Further, the Pak1 gene co-deletion experiment illustrated the potential of such studies to illuminate causal pathogenic pathways, by suggesting functional localisation of the primary Ras-related pathology in the amygdala and other parts of the social brain network, and causal involvement of specific synaptic proteins [7].

Translational statin intervention studies in human NF1, based on the Ras downregulation hypothesis, have had mixed results. Improvements in verbal and non-verbal memory were reported within a 12-week phase 1 single-arm study examining the safety and tolerability of lovastatin in 23 children aged 10–17 years [19] and in a 14-week randomised controlled trial (RCT) of lovastatin in 44 10–50-year-olds [20]. Normalisation of pseudo-resting state functional connectivity in areas of the default mode network (DMN) following lovastatin treatment was found in a case series of 7 children from the prior child cohort [21]. A case-control study of trans-cranial magnetic stimulation in 11 adults with NF1 showed impaired synaptic plasticity and deficits in phasic alertness at baseline compared to controls, which improved after 4 days of high-dose (200 mg) lovastatin [22]. However, larger statin trials have found little effect. A 12-week double-blind, placebo-controlled RCT of simvastatin in 62 children with NF1 aged 8–16 found no group differences on primary behavioural outcome measures and minimal improvements in cognitive aspects of visual synthesis in the simvastatin group; [23] another RCT of simvastatin (84 children aged 8–16 years) found no improvements in cognitive deficits or parent-reported behavioural problems [24]. A 16-week RCT of lovastatin in 146 8–15-year-olds with NF1 and visuospatial learning/attention deficits found no improvements on a paired associate learning task [25].

This human intervention work has been on mid-childhood or older cohorts rather than in earlier development. Trials have also not specifically targeted NF1-autism behavioural outcomes or used multiparametric imaging techniques. We report here therefore on the first experimental trial of a statin in young children with NF1 with co-occurring autism, using detailed multilevel measurements designed to assess the pathogenic pathway identified in NF1 animal models from gene disruption to cognitive and behavioural pathology. These included (i) statin effects at a cellular level on Ras activation, using peripheral MAPKinase assay; (ii) multiparametric imaging to reflect different related aspects of neural system structure, neurophysiology and in vivo function; and (iii) NF1-relevant cognitive and autism-related behavioural outcomes. By investigating statin effects on these different levels, we aim to illuminate the dynamics and possible causal relationships of this pathogenic pathway in humans. Hypotheses were that (i) statin treatment in young children with NF1-autism would be feasible, safe and acceptable to families; (ii) peripheral MAPKinase assay and awake multiparametric imaging could be acquired; and (iii) that although the study was not powered for definitive treatment effect estimation, signals of change in MAPK and multimodal imaging parameters would be detectable, along with change in autism and other cognitive and behavioural symptoms. Specific imaging parameters for testing and hypothesised parameter changes were selected on the basis of the existing imaging literature in NF1, especially those linked to known abnormalities in idiopathic autism. Thus, we expected normalisation of the reduced GABA and perfusion metrics, and reduction in the abnormalities in DTI and connectivity metrics found in NF1 (see “Methods” section).

## Methods

### Design and participants

A single-site triple-blind (clinician, family, assesor) RCT of simvastatin vs. placebo in children with NF1-autism, the SimvAstatin in Neurofibromatosis Type 1-Autism (SANTA) trial, was registered with EudraCT number 2012-005742-38. Study protocol is available on http://research.bmh.manchester.ac.uk/santa. Participants were children between 4.5–10.5 years meeting diagnostic criteria for (i) NF1 (National Institutes of Health criteria) [26] (ii) autism spectrum disorder (ASD) using Collaborative Program of Excellence in Autism (CPEA) criteria, based on Autism Diagnostic Interview-Revised (ADI-R), Autism Diagnostic Observation Scale-2 and WASI (Wechsler Abbreviated Scale of Intelligence) verbal IQ, [27] after positive initial screening (*T* > 60) on parent-rated Social Responsiveness Scale (SRS). They were recruited via local and regional UK NF1 clinics (Manchester, Leeds, Newcastle, Edinburgh, Wirral, Warrington and Edinburgh, UK) and through NF charities’ newsletters, websites and social media pages. Exclusion criteria were (i) severe learning disability (WASI verbal IQ < 50); (ii) in active treatment for another NF1 complication (e.g., chemotherapy for optic pathway or other low-grade glioma, Ilizarov frame for pseudarthrosis) or clinically significant unrelated illness; (iii) abnormal liver function or creatinine kinase at baseline (iv) parents of participants with insufficient English to complete the ASD screening assessments; (v) use of psychotropic medication other than stimulants, current simvastatin use or any investigational drug within 4 months of screening; (vi) participants with planned surgery within 16 weeks of potential enrolment. Participants on a stable dose of stimulants for at least 3 months prior to screening were permitted to participate.

### Measures

MAPK assay (baseline, 12-week endpoint) in peripheral lymphocytes was used as a marker of the effectiveness of statin-induced downregulation of the intracellular Ras pathway. In animal models, peripheral estimation of this kind has shown consistency with neural Ras activity, and in humans, has been associated with cognitive function in Alzheimer’s disease and cognitive impairment [28, 29]. Details of methodology and assay are given in Additional file 1.

Brain imaging (baseline, 12-week endpoint) on a Philips 3T Achieva scanner (Eindhoven, The Netherlands) was implemented using a 32-channel head coil for signal reception and body coil for transmission with no contrast or sedation. Imaging parameters were purposefully selected on the basis of prior hypotheses relating to existing imaging findings in NF1 on cortical GABA spectroscopy [12, 13], MR cerebral perfusion [14], alteration in diffusion-weighted imaging [15] and abnormal network connectivity on resting state fMRI [16, 17]. Additional file 1: Table S1 outlines the imaging protocol, along with our patient preparation protocol to facilitate awake scanning in this challenging imaging cohort. No visual stimulation was allowed for the initial resting state fMRI acquisition, but following this, the children were allowed to watch a projected film of their choice or listen to music if they preferred. Imaging data were acquired at week 0 and then again following either exposure to placebo or simvastatin for 12 weeks. In four cases, where the initial imaging dataset was incomplete, a week 4 scan was performed which acquired only the missing week 0 imaging datasets (T1 volume and diffusion data).

Autism symptoms (baseline, 4 weeks, 12-week endpoint) were quantified using standard measures of proven specificity and sensitivity to treatment effect over short periods and widely used in autism psychopharmacology trials [30, 31]. Parent-rated *Aberrant Behaviour Checklist* (ABC) [32] has 58-items on 1–4 Likert scale with five subscales: irritability, hyperactivity, lethargy/withdrawal, stereotypy and inappropriate speech. *Parent-defined target symptoms* [33] was based on blinded researcher interview. One or two problems of greatest concern to parents at baseline, rated on frequency, duration, intensity and functional impairment, were assessed on a 9-point scale as 1 = normal, 2 = markedly improved, 3 = definitely improved, 4 = equivocally better, 5 = no change, 6 = equivocally worse, 7 = definitely worse, 8 = markedly worse, and 9 = disastrously worse. Ratings across the two target symptoms were averaged. *Clinical Global Impression Scale* (CGI-S) [34] was used in measuring severity of psychopathology on a 7-point scale, change from the initiation of treatment on a similar 7-point scale and the drug efficacy index. Over three decades of research the CGI correlates well with standard research drug efficacy scales [34]. *Overactivity symptoms* were assessed using the standard parent-rated Conners questionnaire [35]. Following standard practice in autism medication trials [36], *clinical responders* were defined as children with 25% reduction in the parent-rated ABC irritability score plus a rating of ‘much improved’ or ‘very much improved’ on the clinician-rated CGI scale.

#### Acceptability

Telephone interviews were conducted at 16 weeks (4 weeks after the end of the trial) by researchers independent of the trial research team, and blind to treatment arm, to assess parent acceptability of the trial protocol. This 19-item interview was rated on a 5-point Likert scale from strongly disagree to strongly agree for each stem statement.

### Procedures

Eligible participants were randomised on a 1:1 ratio by the clinical trial pharmacy at Manchester University NHS Foundation Trust using web-based randomisation with blocks of 2 and 4. The results of the randomisation were not communicated outside the pharmacy, which delivered the appropriate masked drug bottles to the research team. All investigators, participants and their parents were kept masked to treatment allocation.

Simvastatin is an HMG-CoA reductase inhibitor. It has a UK and US licence for use in age 10 and above, and there is extensive off-label clinical experience of its use in younger children with other disorders such as familial hypercholesterolaemia and Smith Lemli Opitz Syndrome. The bioavailability of simvastatin is 42.5% ± 42.5. The only other available statin which crosses the blood brain barrier (lovastatin) is not licenced for use in children in Europe.

Assessments were carried out at the NIHR/Wellcome Trust Clinical Research Facility, Manchester University NHS Foundation Trust at baseline and weeks 4 and 12. After baseline and randomisation, participants were treated with simvastatin or placebo in liquid preparation at 0.5 mg/kg in a single daily dose. At week 4, in the absence of any reported adverse effects, or abnormalities of plasma biochemistry (LFTs and CK), simvastatin dose was increased to 1 mg/kg/day to a maximum of 30 mg/day. This dosing regime was similar to those used in other studies of simvastatin in young children [37] and was selected for known safety and indirectly for known efficacy in such other contexts.

#### Consent and ethics

We obtained informed oral and written consent from parents and assent from children where developmentally appropriate. The local ethics committee approved the study (REC Reference 13/NW/0111). The trial was conducted in agreement with the Declaration of Helsinki and Good Clinical Practice Guidelines.

### Statistical analysis

#### Behavioural measures

Statistical analysis was performed in Stata version 14, based on an intention-to-treat approach using all randomised patients and followed the CONSORT statement and trial protocol. The only protocol measure not presented in this report is the Judgement of Line Orientation Test, for which insufficient analysable data were available (details in Additional file 1). The primary analysis was based on tabulated and associated graphical summaries of feasibility indicators: patient recruitment, checks for the absence of selective recruitment of participants; baseline balance of summary statistics and patient flow. The study was not powered for formal analysis of between-group treatment effect on clinical and behavioural outcomes; the presented results focus on point estimates and associated 95% confidence intervals rather than statistical significance. Analysis was performed using linear regression models to estimate the effect of random allocation on autism and behavioural symptom outcomes at 12 weeks, adjusting for baseline values of the relevant outcome as a linear covariate. Bootstrapping with 500 replications was used to estimate standard errors for all models.

#### Imaging analysis (further details in Additional file 1)

##### GABA spectroscopy

GABA measurements were taken from (i) frontal white matter (FWM) and (ii) deep grey nuclei (DGN) using the localised spectroscopy sequence MEGA-PRESS, using the unsuppressed water signal as a reference. GABA measurement is defined as ‘GABA+’, due to macromolecular signal contribution [38]. The sum of glutamate and glutamine (Glx) was measured via the same acquisition, giving a peak centred at 3.75 ppm. A non-water-suppressed acquisition from the same locations was acquired to act as reference. Statistical analyses in SPSS 22.0 considered the absolute and between-group change from baseline to endpoint, with and without adjustment for baseline variation. Parametric (*t* test) and non-parametric (Mann-Whitney test with covariate adjustment) [39] tests were used for comparison, based on normality of the data. No correction was made for testing across multiple regions.

##### Perfusion imaging

Pulsed arterial spin labelling images were acquired using a modified ‘STAR’ technique [40], together with co-aligned proton density images. Perfusion images were obtained by subtracting control images from labelled images and fitting to a single blood-compartment model using an in-house code provided by LP (see Additional file 1: Table S1). The median regional CBF values were calculated following CBF map registration to the corresponding structural T1 images.

##### Diffusion imaging

We applied a diffusion-weighted multislice spin echo single-shot echo planar imaging sequence transaxially: slices 55 contiguous, *b* = 1000 s/mm^2^ (Δ/δ 36.4/22.7 ms) in 6 non-collinear directions. One volume (b0 image) was also acquired without a diffusion gradient; *b* = 0 s/mm^2^. The median regional ADC values were calculated following ADC map registration to the corresponding structural T1 images.

##### Resting state fMRI

Single-shot, whole brain coverage, echo planar imaging was used to acquire resting state data (Additional file 1: Table S1). Spatial networks demonstrating strong temporal co-activation in the resting BOLD fMRI responses were defined using probabilistic independent component analysis (ICA). The analysis for differences between groups was performed using a dual regression technique, which allowed for voxel-by-voxel comparisons of functional connectivity.

##### Machine learning

The whole imaging dataset was analysed for stratification into simvastatin or placebo groups with a Random Forest machine learning classifier. Cross-validation was performed, such that each fold contained at least one example of each group. The significance of the resulting area under the curve (AUC) score was assessed using a test where the group labels were permuted (Python scikit-learn library) [41].

## Results

### Trial flow

Additional file 1: Figure S4 shows the CONSORT flow chart for the study. Ninety-one completed parent-reported SRS questionnaires were received between October 2013 and June 2015. Of these, 71 met eligibility criteria and were invited for in-depth assessment; 53 were seen for baseline ASD assessments, from which 30 met CPEA criteria for ASD and were randomised (placebo, 16; simvastatin, 14); 26 completed endpoint assessment at 12 weeks. All analyses were by assigned groups.

### Demographics and baseline status

Additional file 1: Tables S2 and S3 shows baseline demographic and clinical data for the two groups. The mean age of the sample was 8.10 years. Two participants in the simvastatin arm and two in the placebo arm had a pre-existing diagnosis of ADHD and were on stimulant medication. Baseline measures including ADI-R, ADOS, Verbal IQ and SRS scores were generally well matched across the groups, and the screening and diagnostic autism measures all showed values well within the standardised autism range (Additional file 1: Table S2). In the simvastatin group, 21.4% had inherited the NF1 mutations as opposed to 62.5% in the placebo group, but we have no evidence of any differential effect from familial or sporadic cases on any baseline or outcome variable from our previous studies [4, 42]. Genotype data on the cohort is presented in Additional file 1: Table S7 where it is also compared to a large recently published genotyped cohort from our group [43]. There are no obvious differences in mutation type between the SANTA cohort and the larger cohort, suggesting representativeness of the SANTA cohort. There was no SPRED1 and only one microdeletion in the cohort. Patient defined target symptoms included hyperactivity, aggression, social inappropriateness, difficulties with communication, inflexibility/obsessionality and learning problems. In four cases (three in simvastatin, one in placebo), there was movement artefact on the T1 volume and diffusion sequences, and these parameters were then re-acquired at week 4 visit.

### Acceptability

Sixteen-week telephone interview data was available for 25 participants. The scanning protocol was acceptable for all of these families, 21/25 families felt that the habituation CD helped with the scanning process.

### Adverse events

Adverse events (AEs) recorded are set out in Additional file 1: Table S4. These were all minor and not specific to the simvastatin arm; none resulted in drug discontinuation or dose reduction. There were no severe adverse events or suspected unexpected serious adverse reactions.

### Outcome estimation

#### Peripheral MAPK activity

**Fig. 1:**
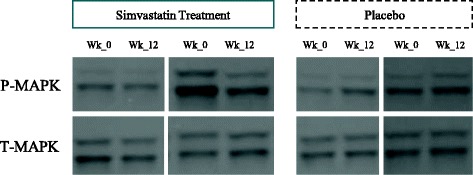
Representative Western blot showing p MAPK (top) and total MAPK (bottom) levels in peripheral blood mononuclear cells from NF1 patient treated either with placebo or simvastatin

**Fig. 2:**
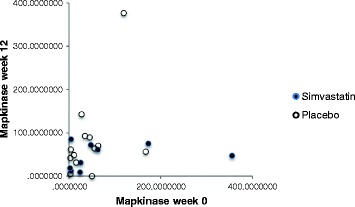
Distribution of MAP Kinase Assay levels at baseline and endpoint

Completed assay was achieved in 27/30 cases (12/14 simvastatin, 15/16 placebo) at baseline and 22/26 (9/11 simvastatin, 13/15 placebo) at endpoint. Missing data is related to inadequate venesection volumes and the need to prioritise adverse event monitoring. Representative Western blot assays are shown in Fig. 1 and quantification of outcomes in Additional file 1: Figure S6. Assay results showed wide variance; robust estimation using a linear method gave a moderate between-group treatment effect size point estimate of 0.60 reduction of pMAPK in favour of intervention, but with 95% CI − .34 to 1.54, ranging from small increase to large reduction (Fig. 2).

#### Imaging

##### MR spectroscopy

**Fig. 3:**
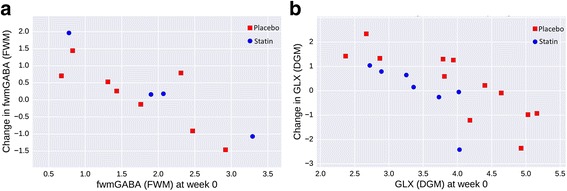
MR spectroscopy; change in **a** frontal white matter (FWM) GABA and **b** deep grey nuclei (DGN) Glx

MRS data was acquired for frontal white matter (FWM) in 27/30 cases at baseline and 19/26 at endpoint. Within this, endpoint voxel assessment of GABA+ data was possible in 5/11 simvastatin and 9/15 placebo and showed a trend towards between-group increase in the simvastatin group compared to placebo (mean 1.82 placebo vs. 2.39 simvastatin (*t*(12) = − 2.12, *p* = .055, two-tailed uncorrected), although this was not present when adjusted for baseline values (ANCOVA *p* = 0.188, Mann-Whitney *p* = 0.66; Fig. 3a). Glx showed no effect, but GABA/Glx ratio showed significant endpoint difference (*t*(12) = − 2.78, *p* = .016 two-tailed, uncorrected). MRS data for deep grey nuclei (DGN) was acquired in 24/30 at baseline and 23/26 at endpoint. Pre-post analysis was possible on 13 simvastatin and 12 placebo; it showed no change in GABA+ value but a significant post-treatment reduction in Glx compared to placebo (ANCOVA *p* < 0.05, Mann-Whitney *p* < 0.01; Fig. 3b), although uncorrected for a significantly lower Glx in the treatment group at baseline (*t*(18) = − 3.08, *p* = .006).

##### Perfusion and diffusivity assessment

**Fig. 4:**
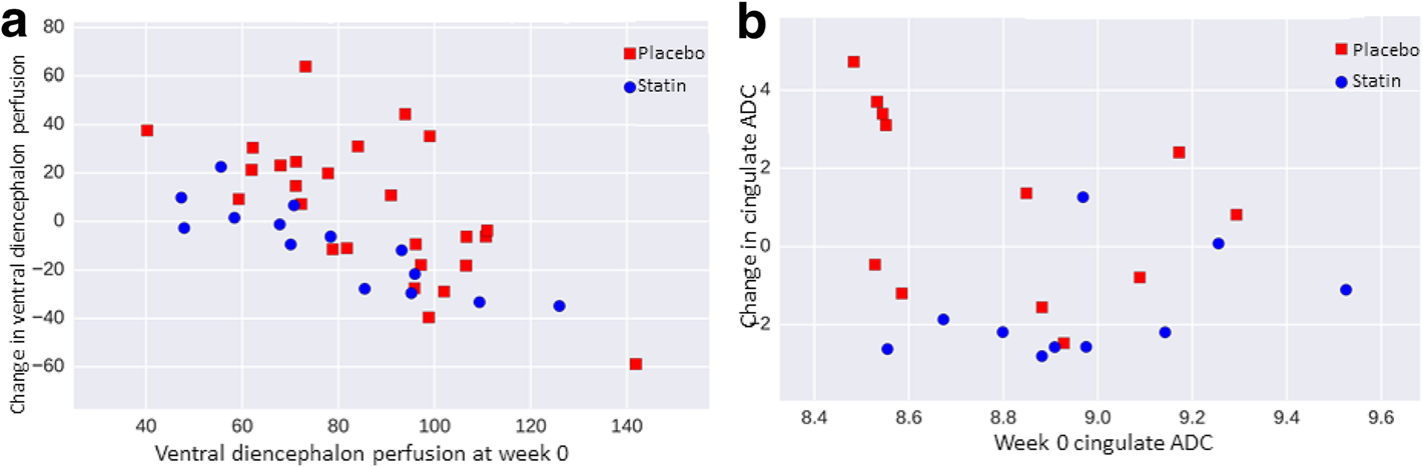
**a** Change in perfusion measured from ASL in the ventral diencephalon and **b** changes in ADC value in the cingulate cortex

Validated data for diffusion analysis were acquired on 20/30 cases at baseline (10/14 simvastatin, 10/16 placebo) and 16/26 at endpoint (8/11 simvastatin, 8/15 placebo). Perfusion data analysis was available on 28/30 cases at baseline (12/14 simvastatin cases and 14/16 placebo) and 23/30 cases post treatment (10/14 simvastatin and 13/16 placebo). Analysis of available paired pre-post data (7 simvastatin and 13 placebo) showed significant increase in perfusion within the ventral diencephalon associated with statin treatment (ANCOVA *p* < 0.01 and Mann-Whitney *p* < 0.01, uncorrected; Fig. 4a). Analysis of available paired pre-post data (5 simvastatin and 6 placebo) showed decrease in ADC within the cingulate gyrus associated with statin treatment (ANCOVA *p* = 0.01, Mann-Whitney *p* < 0.01, uncorrected; Fig. 4b).

##### Resting state fMRI

**Fig. 5:**
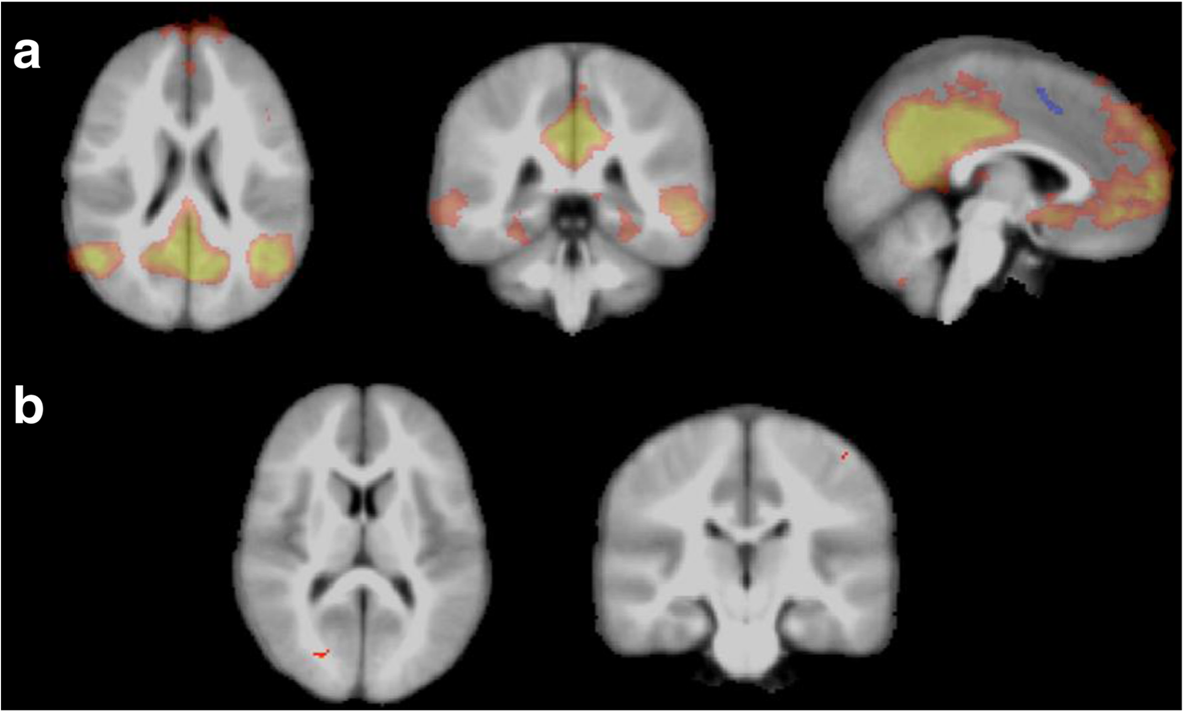
rsfMRI **a** default mode network (DMN) demonstrated by probabilistic group ICA of week 12 acquisitions (axial, coronal, sagittal). **b** At week 12, foci of decreased DMN co-activation were identified at the 10% level within the right occipital and left perirolandic regions. No significant differences in the DMN were identified at the 5% level, corrected

Probabilistic ICA identified the default mode network (DMN) separately in both baseline (10/14 simvastatin, 11/16 placebo) and week 12 (6/11 simvastatin, 11/15 placebo) rsfMRI acquisitions. Dual regression did not identify any significant differences between simvastatin and placebo groups in the DMN spatial maps when tested at the 5% significance level (corrected for multiple comparisons). However, at the 10% significance level foci of decreased co-activation in the simvastatin group compared to placebo were seen within the right occipital lobe and left perirolandic region (*p* = 0.093 and 0.092, respectively, corrected, voxel counts 11 and 3; Fig. 5). No significant differences were seen at the 10% level in the DMN at week zero nor in the sensorimotor or medial visual networks at either time point.

##### Machine learning

**Fig. 6:**
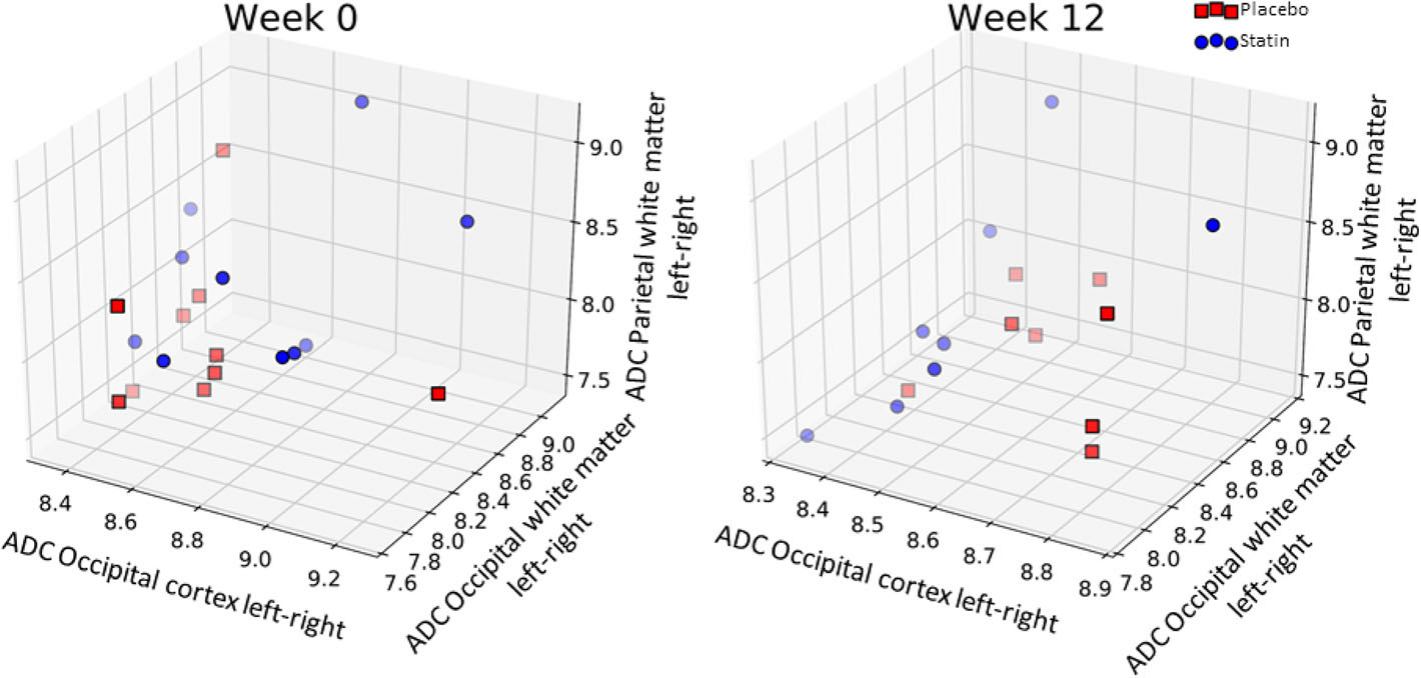
Three-dimensional plot of the ADC values in the occipital cortex, parietal and occipital white matter; right vs. left

The whole imaging dataset was entered for analysis. Baseline classification accuracy was 64% (*p* = 0.25) compatible with stratification into groups on the basis of chance alone. Following treatment, the features with best statistical power for group allocation were the ADC values in the occipital cortex, the occipital white matter and the parietal white matter. We compared changes in the left- and right-sided ADC metric in these regions between both groups and found classification accuracy rose from baseline to 79% (*p* < 0.05; Fig. 6), suggesting a simvastatin treatment effect.

#### Behavioural outcomes

Behavioural symptom endpoint outcomes are shown in Table 1 (and week 4 intermediate outcomes in Additional file 1: Table S5). The trial was not powered to show significant between-group behaviour effects, and none were seen. At endpoint*,* 3/12 (25%) of the statin treatment cases were classified as clinical responders using standard RUPP (Research Units of Paediatric Psychopharmacology) criteria [26] compared to 0/14 (0%) in the placebo group. Each of these responders also met subsidiary standards for response on the patient-defined target symptoms (PDTS < 3). Two further cases in the statin group and 2/14 in the placebo group met PDTS responder criteria only. The responder group (*n* = 3) was characterised clinically by being male, with the mean age of 9.29 years (SD 0.77), with a relatively high-baseline ADOS total score of 17.0 (SD 1.73) but other metrics similar to group means (baseline SRS total score = 87.6 (SD 2.08), ADI-R social interaction 20.66 (SD 1.52), communication 15 (SD1.73) and RRBs 5.33 (1.55).

**Table 1:** Endpoint behavioural outcomes

Week 12 outcomes	Summary statistics			Mean difference			
	Sample	Placebo	Simvastatin	Adjusted mean difference (95% CI)	Bootstrap SE	Effect size (95% CI)	Number analysed
ABC	*N* = 28	*N* = 15	*N* = 13				
Irritability	19.14 (11.62)	16.40 (10.82)	22.31 (12.14)	1.66 (− 4.61, 7.93)	3.20	0.14 (− 0.40, 0.68)	28
Lethargy^*^	12.63 (10.01)	10.53 (9.61)	15.25 (10.30)^*^	3.60 (− 4.09, 11.28)	3.92	0.29 (− 0.41, 1.13)	26
Stereotypy	5.71 (5.08)	3.93 (3.63)	7.77 (5.83)	1.61 (− 0.98, 4.20)	1.32	0.32 (− 0.19, 0.83)	28
Hyperactivity	23.61 (13.68)	19.13 (13.17)	28.77(12.83)	3.87 (− 3.28, 11.01)	3.65	0.28 (− 0.24, 0.80)	28
Inappropriate speech	5.89 (3.11)	4.80 (2.54)	7.15 (3.31)	1.77 (− 0.10, 3.63)	0.95	0.57 (− 0.03, 1.17)	28
25% reduction irritability subscale	*N* = 11	*N* = 5	*N* = 6				
Conners	*N* = 28	*N* = 15	*N* = 13				
Inattention	77.25 (12.57)	74.53 (14.16)	80.38 (10.08)	5.33 (− 0.96, 11.61)	3.21	0.42 (− 0.08, 0.92)	27
Hyperactivity	73.54 (15.92)	69.40 (17.12)	78.31 (13.51)	−0.98 (− 8.09, 6.13)	3.63	−0.06 (− 0.51, 0.39)	27
Learning problems	69.25 (14.62)	65.40 (12.91)	73.69 (15.71)	1.59 (− 2.13, 5.30)	1.89	0.11 (− 0.15, 0.36)	27
Executive function	71.82 (14.59)	68.20 (16.24)	76.00 (11.66)	4.04 (− 2.51, 10.59)	3.34	0.28 (− 0.17, 0.73)	27
Aggression	70.43 (18.98)	68.40 (20.86)	72.77 (17.09)	− 0.05 (− 11.05,10.96)	5.61	− 0.00 (− 0.58, 0.58)	27
Peer relations	83.89 (11.53)	82.00 (13.51)	86.08 (8.73)	1.38 (− 4.45, 7.21)	2.97	0.12 (− 0.39, 0.63)	27
Parent-defined target symptoms (PDTS)	*N* = 26	*N* = 14	*N* = 12				
Mean (SD)	3.52 (1.77)	3.75 (1.86)	3.25 (1.68)				
Responders (PDTS score < 3)	7	2	5				
CGI^*^	*N* = 26	*N* = 14	*N* = 12				
Global improvement Mean (SD)	3.31 (0.84)	3.57 (0.85)	3.00 (0.74)				
Treatment responder+	*N* = 3	*N* = 0	*N* = 3				

^*^1 additional observation missing. +Treatment responder defined as > 25% reduction in ABC irritability subscale and a score of improved or much improved on CGI

Higher scores on ABC, Conners and CGI are indicative of higher levels of impairment

## Discussion

Previous statin trials in older children and adults have shown mixed effects, but most used lovastatin and measured outcomes at just cognitive or behavioural levels. This trial used simvastatin, considered the most effective neuroprotective statin [44]. It is also the first trial that has looked in detail at statin effects on ‘upstream’ process at cell and neural system levels, reflecting a pathogenic pathway between gene disruption and the autism-related behavioural psychopathological outcomes known in NF1. We interpret the outcomes therefore in relation to each of these levels, while acknowledging that the restricted sample size in this data-rich trial, and the variable amounts of data available for different analyses, limits precision of estimation.

At the *cellular level*, the moderate between-group point estimate showing peripheral lymphocyte reduction of MAPK function was in the hypothesised direction, consistent with a statin effect at the cell level on the Ras pathway activation; the wide 95% CI values ranged from a large decrease to a small increase. Preparation, international transport and storage of the samples may have introduced increased variance in the assay results.

At a *neural system level*, neuroimaging shows evidence of specific statin effects in regions of interest of the brain including frontal white matter, deep grey nuclei (lentiform, caudate and thalamic nuclei), cingulate gyrus, ventral diencephalon and occipital/occipito-parietal cortex. Detection of GABA in white matter has been reported in other studies, albeit at lower levels than in grey matter [45]. The effects of the statin on the multiparametric data are in a direction consistent with normalising many aspects of the underlying NF1-related neuropathology identified in previous studies. Thus, the suggested increased absolute GABA levels in frontal white matter is consistent with reversing the reduced cortical GABA found in previous studies in children and young adults with NF1 [12, 13] (a contrast to the increase found in animal experiments [7, 18]). The variation in GABA findings by brain region in our study is echoed in a recent NF1 animal study, [46] reporting differential localization of GABA between prefrontal cortex and hippocampus and speculating that this may relate to differential effects on pre- and post-synaptic receptors. In the future, it would be possible to study this important variability further in humans by measuring GABA type A receptor binding using [^11^C]-flumazenil PET alongside GABA concentration with MRS, as in [12].

Interpretation of our evidence suggesting reduced Glx concentration in deep grey nuclei in relation to the existing NF1 literature is uncertain since findings on Glx concentration in NF1 have been previously conflicting. However, in young children with idiopathic autism, elevated deep brain Glx has been found in the anterior cingulate cortex in one large sample study [47] and reported to correlate with quality of social interaction in another [48]. The finding in this current study therefore can be interpreted within this context as positive in relation to autism symptoms.

The reduction in ADC found within the cingulate gyrus, and the significant ADC finding within the machine learning analysis, needs to be interpreted in the context of other work, which has shown increased ADC and decreased FA values in NF1 including in the cingulate [15]. Such findings suggest reduction in cellular packing and intra-myelinic oedema and have been associated with NF1 neurological symptom status [11]. The effects found in this current study therefore are consistent with reduced extra-cellular water free diffusion in NF1, and a positive simvastatin effect to reduce intra-myelinic oedema and improve cellular packing. The presence of microstructural abnormalities, reflected in increased ADC values, have also been described beyond NF1 in idiopathic autism [49–51] and potentially give these findings wider relevance in relation to this NF1-autism cohort.

The increased perfusion in the ventral diencephalon can be understood in the context of diminished perfusion in cingulate gyrus, medial frontal cortex, centrum semiovale, thalamus and temporo-occipital cortex found in NF1 children (*n* = 14, mean age = 10.2 years) [14] and related hypo-metabolism predominantly within the thalamus in FDG PET studies [52–54]. Statins may increase cerebral blood flow by improving cerebral vasomotor reactivity through increased NO bioavailability, promotion of microvascular reperfusion, and enhanced eNOS in the thalamus, as well as cerebellum, visual cortex and posterior cingulate [55].

No statistically robust difference in the DMN was identified between treatment and placebo groups, but findings at the 10% level raise the possibility of a trend that might be detected in a larger study. Diminished functional connectivity has been found in the posterior cingulate in human NF1 [16], and there is evidence from a small case series with children that statin treatment can induce improvements in functional connectivity in posterior cingulate cortex [21]. Here, simvastatin could potentially be acting in a focal manner on microstructural and vascular changes resulting in better regulation of function through a regional improvement in myelination and resultant neuronal function.

For *behavioural outcomes*, while the sample was too small for definitive estimation, we found that 25% of the simvastatin sample, compared to none of the placebo group, showed a clinical response using standard criteria measured using independently triangulated parent-report with clinician judgement.

### Limitations

Dosing of simvastatin in this study was based on safety and efficacy evidence from use of statins in other human disease contexts; we do not know how appropriate it might be for effectiveness in this context. Animal work showed phenotypic rescue [12] with lovastatin at doses equivalent to those commonly prescribed for children (AJS, data not shown); however, differences in mode of delivery (intraperitoneal in animal studies) and the relative brain penetration of the statins (much higher in simvastatin) make direct comparison between the animal and human studies not meaningful. A valuable next step in this context would be further pre-clinical dose-finding studies in animal models using both statins with a mode of administration comparable to that in humans. Our treatment study was relatively short term, and we cannot generalise in relation to any longer term effects. There is no controlled data as yet to confirm a specific link between peripheral pMAPK assay and neural Ras function in human NF1 (although links have been found in cognitive impairment and Alzheimer’s disease); further work will be necessary to confirm its value as a biomarker. Due to the technical challenges of imaging children with developmental disability at this age, the amount of analysable data varied for each imaging parameter. The study was not powered for a formal test of effectiveness; inferences on statin effects are preliminary and serve to indicate hypotheses and outcomes of interest for future larger scale work.

## Conclusions

This study demonstrates the acceptability and safety of simvastatin treatment for young children with NF1 and autism; feasibility of awake scanning, data acquisition and peripheral biomarker assay in such children given the right preparation; and the value of such a multiparametric approach in capturing the likely complexity of pathogenic mechanisms.

The trial findings are suggestive of specific simvastatin effects in brain areas that have been shown to be part of NF1 neural pathology in previous studies. Furthermore, many of these areas have functional significance as part of the ‘social brain network’, highly associated with social impairment and autism psychopathology [56]. This functional localisation may thus be relevant both to the high autism prevalence in NF1 and to how simvastatin could have specific remedial effects on NF1-autism at the level of brain structure and function.

In terms of pathophysiological mechanism, the initial rationale for statin intervention was its action in NF1 animal models to downregulate the Ras pathway with consequent effect to reduce GABA, improve synaptic long-term potentiation and rescue the behavioural phenotype [7, 8, 18]. This trial in young children gives evidence consistent with that model operating in humans through its evidence of a simvastatin treatment effect (albeit with wide CI) towards reduced cellular pMAPK activation on peripheral assay, and associated biologically plausible effects found on GABA/glutamate balance in FWM and DGN. However, the results also suggest simvastatin action through additional mechanisms, such as direct effects on myelin formation and regional axonal and astrocyte integrity in NF1. Pleiotropic effects of this kind from statins in the CNS are well recognised [57–59]. Our findings further suggest that treatment may affect such mechanisms in relevant functional brain areas in NF1 autism. This has future potential for insights into causal pathogenesis in autism and NF1 as well as suggesting more focused treatment targets. Larger studies will be necessary to further test these possibilities and to link them to any confirmed effect on behavioural symptom outcomes. While the initial results are encouraging and suggest specific hypotheses for further testing, this preliminary study was not powered to provide evidence to support clinical use of simvastatin in the disorder in children at this time.

In a wider context, the SANTA trial is, to our knowledge, the first RCT in syndromic autism, or indeed in clinical neuroscience generally, to have successfully tested effects simultaneously on relevant cellular activity markers, neural system multiparametric imaging and behavioural outcomes. As such, it provides a model of a new cohort of experimental intervention designs to link brain process and behavioural outcomes in the context of an experimental intervention trial. This has the eventual goal of treatment discovery in autism, plus the illumination of pathogenic pathways from gene effect to behavioural outcome in neuropsychiatric disorder; in terms of  both regional brain localization and underlying pathogenic mechanisms.

## Additional file

Additional file 1: **Supplementary Materials. Table S1.** MRI sequence parameters and scan time duration for a complete imaging acquisition lasting approximately 45 min (including scout sequences and planning time). **Table S2.** Baseline descriptive data. **Table S3.** Baseline clinical findings. **Table S4.** Adverse events. **Table S5.** Week 4 intermediate outcomes. **Table S6.** Quantification of MAPK outcomes at baseline and endpoint. **Table S7.** A comparison of the mutation data in the SANTA sample to previously reported data from a clinic referred NF1 sample (see text). **Figure S1.** a) Spectrum obtained from 3 × 3 × 3 voxel placed in deep grey matter of a 5-year-old child using MEGA-PRESS suppression scheme at 3T (top, non-edited subspectrum; bottom, GABA-edited spectrum) showing signals from amino-acid protons (AA), choline-containing compounds (cho), creatine + phosphocreatine (cr), N-acetylaspartate (NAA), GABA and glutamate + glutamine (Glx). b) Figure depicting example output of AMARES Model fitting in jMRUI. **Figure S2.** Example locations of VOI (3 × 3 × 3 cm^3^) acquired from a) left fontal white matter and b) deep grey matter (including caudate, lentiform nucleus, thalamus and putamen). **Figure S3.** Example illustrating in sagittal view the position of the perfusion-imaging slices, which were planed above the ventricles and the labelling slab (150 mm) that was set 10 mm below the imaging slices. **Figure S4.** SANTA CONSORT flow diagram. (DOCX 914 kb)

## Abbreviations

ABC: Aberrant Behaviour Checklist
ADC: Apparent Diffusion Coefficient
ADI-R: Autism Diagnostic Interview Revised
ADOS: Autism Diagnostic Observation Schedule
ASD: Autism spectrum disorder
CGI: Clinical Global Impression
DMN: Default mode network
DTI: Diffusion tensor imaging
MRS: Magnetic resonance spectroscopy
NF1: Neurofibromatosis 1
PDTS: Parent-defined target symptoms
SRS: Social Responsiveness Scale
WASI: Wechsler Abbreviated Scale of Intelligence

## References

[1] Evans D, Howard E, Giblin C, Clancy T, Spencer H, Huson S (2010). Birth incidence and prevalence of tumor-prone syndromes: estimates from a UK family genetic register service. Am J Med Genet.

[2] Hyman S, Shores A, North K (2007). The nature and frequency of cognitive deficits in children with neurofibromatosis type 1. Dev Med Child Neurol.

[3] Mautner V, Kluwe L, Thakker S, Leark R (2002). Treatment of ADHD in neurofibromatosis type 1. Dev Med Child Neurol.

[4] Garg S, Green J, Leadbitter K, Emsley R, Lehtonen A, Evans DG (2013). Neurofibromatosis type 1 and autism spectrum disorder. Pediatrics.

[5] Plasschaert E, Descheemaeker MJ, Van Eylen L, Noens I, Steyaert J, Legius E. Prevalence of autism spectrum disorder symptoms in children with neurofibromatosis type 1. Am J Med Genet B Neuropsychiatr Genet. 2014; 10.1002/ajmg.b.32280 25388972

[6] Morris SM, Acosta MT, Garg S, Green J, Huson S, Legius E (2016). Disease burden and symptom structure of autism in neurofibromatosis type 1: a study of the International NF1-ASD Consortium Team (INFACT). JAMA Psychiatry.

[7] Molosh AI, Johnson PL, Spence JP, Arendt D, Federici LM, Bernabe C (2014). Social learning and amygdala disruptions in Nf1 mice are rescued by blocking p21-activated kinase. Nat Neurosci.

[8] Cui Y, Costa RM, Murphy GG, Elgersma Y, Zhu Y, Gutmann DH (2008). Neurofibromin regulation of ERK signaling modulates GABA release and learning. Cell.

[9] Ishii A, Furusho M, Dupree JL, Bansal R (2016). Strength of ERK1/2 MAPK activation determines its effect on myelin and axonal integrity in the adult CNS. J Neurosci.

[10] Mayes DA, Rizvi TA, Titus-Mitchell H, Oberst R, Ciraolo GM, Vorhees CV (2013). Nf1 loss and Ras hyperactivation in oligodendrocytes induce NOS-driven defects in myelin and vasculature. Cell Rep.

[11] Ertan G, Zan E, Yousem DM, Ceritoglu C, Tekes A, Poretti A (2014). Diffusion tensor imaging of neurofibromatosis bright objects in children with neurofibromatosis type 1. Neuroradiol J.

[12] Violante IR, Patricio M, Bernardino I, Rebola J, Abrunhosa AJ, Ferreira N (2016). GABA deficiency in NF1: a multimodal [11C]-flumazenil and spectroscopy study. Neurology.

[13] Violante IR, Ribeiro MJ, Edden RA, Guimaraes P, Bernardino I, Rebola J (2013). GABA deficit in the visual cortex of patients with neurofibromatosis type 1: genotype-phenotype correlations and functional impact. Brain.

[14] Yeom KW, Lober RM, Barnes PD, Campen CJ (2013). Reduced cerebral arterial spin-labeled perfusion in children with neurofibromatosis type 1. AJNR Am J Neuroradiol.

[15] Karlsgodt KH, Rosser T, Lutkenhoff ES, Cannon TD, Silva A, Bearden CE (2012). Alterations in white matter microstructure in neurofibromatosis-1. PLoS One.

[16] Tomson SN, Schreiner MJ, Narayan M, Rosser T, Enrique N, Silva AJ (2015). Resting state functional MRI reveals abnormal network connectivity in neurofibromatosis 1. Hum Brain Mapp.

[17] Loitfelder M, Huijbregts SC, Veer IM, Swaab HS, Van Buchem MA, Schmidt R (2015). Functional connectivity changes and executive and social problems in neurofibromatosis type I. Brain Connectivity.

[18] Li W, Cui Y, Kushner SA, Brown RA, Jentsch JD, Frankland PW (2005). The HMG-CoA reductase inhibitor lovastatin reverses the learning and attention deficits in a mouse model of neurofibromatosis type 1. Curr Biol.

[19] Acosta M, Kardel P, Walsh K, Rosenbaum K, Gioia G, Packer R (2011). Lovastatin as treatment for neurocognitive deficits in neurofibromatosis type 1: phase 1 study. Pediatr Neurol.

[20] Bearden CE, Hellemann GS, Rosser T, Montojo C, Jonas R, Enrique N (2016). A randomized placebo-controlled lovastatin trial for neurobehavioral function in neurofibromatosis I. Ann Clin Transl Neurol.

[21] Chabernaud C, Mennes M, Kardel P, Gaillard W, Kalbfleisch L, VanMeter J (2012). Lovastatin regulates brain spontaneous low-frequency brain activity in neurofibromatosis type 1. Neurosci Lett.

[22] Mainberger F, Jung NH, Zenker M, Wahllander U, Freudenberg L, Langer S (2013). Lovastatin improves impaired synaptic plasticity and phasic alertness in patients with neurofibromatosis type 1. BMC Neurol.

[23] Krab L, de Goede-Bolde RA, Aarsen F, Pluijm S, Bouman M, van der Geest J (2008). Effect of simvastatin on cognitive functioning in children with neurofibromatosis type 1: a randomized controlled trial. JAMA.

[24] van der Vaart T, Plasschaert E, Rietman AB, Renard M, Oostenbrink R, Vogels A (2013). Simvastatin for cognitive deficits and behavioural problems in patients with neurofibromatosis type 1 (NF1-SIMCODA): a randomised, placebo-controlled trial. Lancet Neurol.

[25] Payne JM, Barton B, Ullrich NJ, Cantor A, Hearps SJ, Cutter G (2016). Randomized placebo-controlled study of lovastatin in children with neurofibromatosis type 1. Neurology.

[26] National Institutes of Health Consensus Development Conference (1988). Neurofibromatosis conference statement. Arch Neurol.

[27] Lainhart JE, Bigler ED, Bocian M, Coon H, Dinh E, Dawson G (2006). Head circumference and height in autism: a study by the Collaborative Program of Excellence in Autism. Am J Med Genet A.

[28] Kayano M, Higaki S, Satoh JI, Matsumoto K, Matsubara E, Takikawa O (2016). Plasma microRNA biomarker detection for mild cognitive impairment using differential correlation analysis. Biomarker Res.

[29] Kiddle SJ, Steves CJ, Mehta M, Simmons A, Xu X, Newhouse S (2015). Plasma protein biomarkers of Alzheimer’s disease endophenotypes in asymptomatic older twins: early cognitive decline and regional brain volumes. Transl Psychiatry.

[30] Sandler A, Sutton K, DeWeese J, Girardi M, Sheppard V, Bodfish J (1999). Lack of benefit of a single dose of synthetic human secretin in the treatment of autism and pervasive developmental disorders. N Engl J Med.

[31] Berry-Kravis E, Sumis A, Hervey C, Nelson M, Porges S, Weng N (2008). Open-label treatment trial of lithium to target the underlying defect in fragile X syndrome. J Dev Behav Pediatr.

[32] Aman M (1994). Aberrant behaviour checklist––community.

[33] Arnold LE, Vitiello B, McDougle C, Scahill L, Shah B, Gonzalez NM (2003). Parent-defined target symptoms respond to risperidone in RUPP autism study: customer approach to clinical trials. J Am Acad Child Adolesc Psychiatry.

[34] Leucht S, Engel RR (2006). The relative sensitivity of the Clinical Global Impressions Scale and the Brief Psychiatric Rating Scale in antipsychotic drug trials. Neuropsychopharmacology.

[35] Conners K, Sitarenios G, Parker J, Epstein J (1998). The revised Conners’ parent rating scale (CPRS-R): factor structure, reliability, and criterion validity. J Abnorm Child Psychol.

[36] McCracken JT, McGough J, Shah B, Cronin P, Hong D, Aman MG (2002). Risperidone in children with autism and serious behavioral problems. N Engl J Med.

[37] Haas D, Garbade SF, Vohwinkel C, Muschol N, Trefz FK, Penzien JM (2007). Effects of cholesterol and simvastatin treatment in patients with Smith-Lemli-Opitz syndrome (SLOS). J Inherit Metab Dis.

[38] Mullins PG, McGonigle DJ, O'Gorman RL, Puts NA, Vidyasagar R, Evans CJ (2014). Current practice in the use of MEGA-PRESS spectroscopy for the detection of GABA. NeuroImage.

[39] Vermeulen K, Thas O, Vansteelandt S (2015). Increasing the power of the Mann-Whitney test in randomized experiments through flexible covariate adjustment. Stat Med.

[40] Petersen ET, Lim T, Golay X (2006). Model-free arterial spin labeling quantification approach for perfusion MRI. Magn Reson Med.

[41] Pedregosa F, Varoquaux G, Gramfort A, Michel V, Thirion B, Grisel O (2011). Scikit-learn: machine learning in python. J Mach Learn Res.

[42] Garg S, Lehtonen A, Huson SM, Emsley R, Trump D, Evans DG (2013). Autism and other psychiatric comorbidity in neurofibromatosis type 1: evidence from a population-based study. Dev Med Child Neurol.

[43] Evans DG, Bowers N, Burkitt-Wright E, Miles E, Garg S, Scott-Kitching V (2016). Comprehensive RNA analysis of the NF1 gene in classically affected NF1 affected individuals meeting NIH criteria has high sensitivity and mutation negative testing is reassuring in isolated cases with pigmentary features only. EBioMed.

[44] Sierra S, Ramos MC, Molina P, Esteo C, Vazquez JA, Burgos JS (2011). Statins as neuroprotectants: a comparative in vitro study of lipophilicity, blood-brain-barrier penetration, lowering of brain cholesterol, and decrease of neuron cell death. J Alzheimers Dis.

[45] Mikkelsen M, Singh KD, Brealy JA, Linden DE, Evans CJ (2016). Quantification of gamma-aminobutyric acid (GABA) in 1H MRS volumes composed heterogeneously of grey and white matter. NMR Biomed.

[46] Goncalves J, Violante IR, Sereno J, Leitao RA, Cai Y, Abrunhosa A (2017). Testing the excitation/inhibition imbalance hypothesis in a mouse model of the autism spectrum disorder: in vivo neurospectroscopy and molecular evidence for regional phenotypes. Mol Autism.

[47] Ito H, Mori K, Harada M, Hisaoka S, Toda Y, Mori T (2017). A proton magnetic resonance spectroscopic study in autism spectrum disorder using a 3-tesla clinical magnetic resonance imaging (MRI) system: the anterior cingulate cortex and the left cerebellum. J Child Neurol.

[48] Doyle-Thomas KA, Card D, Soorya LV, Wang AT, Fan J, Anagnostou E (2014). Metabolic mapping of deep brain structures and associations with symptomatology in autism spectrum disorders. Res Autism Spectr Disord.

[49] Sundaram SK, Kumar A, Makki MI, Behen ME, Chugani HT, Chugani DC (2008). Diffusion tensor imaging of frontal lobe in autism spectrum disorder. Cereb Cortex.

[50] Ben Bashat D, Kronfeld-Duenias V, Zachor DA, Ekstein PM, Hendler T, Tarrasch R (2007). Accelerated maturation of white matter in young children with autism: a high b value DWI study. NeuroImage.

[51] Mengotti P, D’Agostini S, Terlevic R, De Colle C, Biasizzo E, Londero D (2011). Altered white matter integrity and development in children with autism: a combined voxel-based morphometry and diffusion imaging study. Brain Res Bull.

[52] Kaplan AM, Chen K, Lawson MA, Wodrich DL, Bonstelle CT, Reiman EM (1997). Positron emission tomography in children with neurofibromatosis-1. J Child Neurol.

[53] Balestri P, Lucignani G, Fois A, Magliani L, Calistri L, Grana C (1994). Cerebral glucose metabolism in neurofibromatosis type 1 assessed with [18F]-2-fluoro-2-deoxy-D-glucose and PET. J Neurol Neurosurg Psychiatry.

[54] Buchert R, von Borczyskowski D, Wilke F, Gronowsky M, Friedrich RE, Brenner W (2008). Reduced thalamic 18F-flurodeoxyglucose retention in adults with neurofibromatosis type 1. Nucl Med Commun.

[55] Beason-Held L, Thambisetty M, Kraut M, Ferruci L, Elkins W, Zonderman A (2012). Longitudinal changes in brain function related to statin use. Alzheimers Dement.

[56] Johnson MH, Griffin R, Csibra G, Halit H, Farroni T, de Haan M (2005). The emergence of the social brain network: evidence from typical and atypical development. Dev Psychopathol.

[57] Liao JK, Laufs U (2005). Pleiotropic effects of statins. Annu Rev Pharmacol Toxicol.

[58] Ling Q, Tejada-Simon MV (2016). Statins and the brain: new perspective for old drugs. Prog Neuro-Psychopharmacol Biol Psychiatry.

[59] Malfitano AM, Marasco G, Proto MC, Laezza C, Gazzerro P, Bifulco M (2014). Statins in neurological disorders: an overview and update. Pharmacol Res.

