# Supplementary material for: Randomised controlled trial of simvastatin treatment for autism in young children with neurofibromatosis type 1 (SANTA)
Source: Mol Autism. 2018 Feb 22;9:12. doi: 10.1186/s13229-018-0190-z (PMC5824534; doi:10.1186/s13229-018-0190-z)
Supplement: Supplementary file 1 — Supplementary Materials. Table S1. MRI sequence parameters and scan time duration for a complete imaging acquisition lasting approximately 45 min (including scout sequences and planning time). Table S2. Baseline descriptive data. Table S3. Baseline clinical findings. Table S4. Adverse events. Table S5. Week 4 intermediate outcomes. Table S6. Quantification of MAPK outcomes at baseline and endpoint. Table S7. A comparison of the mutation data in the SANTA sample to previously reported data from a clinic referred NF1 sample (see text). Figure S1. a) Spectrum obtained from 3 × 3 × 3 voxel placed in deep grey matter of a 5-year-old child using MEGA-PRESS suppression scheme at 3T (top, non-edited subspectrum; bottom, GABA-edited spectrum) showing signals from amino-acid protons (AA), choline-containing compounds (cho), creatine + phosphocreatine (cr), N-acetylaspartate (NAA), GABA and glutamate + glutamine (Glx). b) Figure depicting example output of AMARES Model fitting in jMRUI. Figure S2. Example locations of VOI (3 × 3 × 3 cm3) acquired from a) left fontal white matter and b) deep grey matter (including caudate, lentiform nucleus, thalamus and putamen). Figure S3. Example illustrating in sagittal view the position of the perfusion-imaging slices, which were planed above the ventricles and the labelling slab (150 mm) that was set 10 mm below the imaging slices. Figure S4. SANTA CONSORT flow diagram. (DOCX 914 kb) [file 13229_2018_190_MOESM1_ESM.docx]

**RANDOMISED CONTROLLED TRIAL OF SIMVASTATIN TREATMENT FOR AUTISM IN YOUNG CHILDREN WITH NEUROFIBROMATOSIS TYPE 1 (SANTA). Stivaros et al.**

**Additional file 1**

**Details of analytic techniques**

***MAPK Assay*** Peripheral Blood Mononuclear Cells (PBMCs) were isolated from 10ml venous blood samples and spun into freeze-dried cell pellets (Manchester Biobank). Dried cell pellets were shipped with dry ice to UCLA for analysis using western blot electrophoresis. Protein concentration was determined using a BCA assay kit and equal amounts of proteins (10 ug) were separated by electrophoresis on 4-12% SDS-PAGE gels. After transferring to nitrocellulose membrane, membranes were blocked with 5% BSA in TBS-T for 1 hour then hybridized with a primary antibody (p-MAPK) overnight at 4^0^C. After washing with TBS-T, the membrane was incubated with a secondary antibody in 5% nonfat milk and TBS-T for 1 hour. Signals were visualized by ECL. After detecting p-MAPK, the membranes were striped and re-probed with a total MAPK antibody. The total MAPK were used to normalize with each sample. The following primary antibodies were used: p-MAPK (#9101S, Cell Signaling, 1:1,000), anti-total MAPK (#9102S, Cell Signaling, 1:5,000)

***Imaging preparation*** Participating families were provided with a two-week imaging habituation protocol and an audio CD of study-specific scanner noise. The patient cohort was acclimatised to awake MRI scanning using a social story preparation and two weeks of graded exposure to mp3 recordings of the sounds made by the MRI scanner. This was initially at any time of the day, then specifically at bedtime. These recordings were prioritised in the same order as each specific scan sequence in the imaging protocol. Children and families were assisted through the scanning procedure with a clinical protocol led by a play specialist, clinical nurses and psychiatrists.

***Details of imaging protocol***

**Table S1:** *MRI sequence parameters and scan time duration for a complete imaging acquisition lasting approximately 45 minutes**

| **Sequence Order** | **Sequence Name** | **Duration (min)** | **Parameters** |
| --- | --- | --- | --- |
| 1 | Resting State fMRI with Echo planar imaging (EPI) readout | 8.25 | FOV 224mm, slice thickness 3mm, Slices 42, TE 35ms, TR 3000ms, Flip angle 90^o^ degrees, number of dynamic scans 160, acq matrix 80×76, recon matrix 80. |
| 2 | T1 Inversion-recovery fast gradient echo 3-D volume | 5.75 | FOV 240mm, Voxel size 1x1x1mm^3^, TR 6.9ms, TE 3.2ms, flip angle 9^o^, acq matrix 240×188, recon matrix 240. |
| 3 | T2 Axial Turbo Spin Echo | 1.77 | FOV 230mm, Slice thickness 4mm, slices 28, slice gap 1mm, TE 80ms, TR 3000ms, acq matrix 400×255, recon matrix 512. |
| 4 | Diffusion Tensor Imaging with EPI readout | 1.87 | FOV 230mm, Slice thickness 2.5mm, slices 55, b value 0 and 1000 smm^-2^, Directions 6, TR 5353ms, TE 73ms, slice gap 0, acq matrix 112×86, recon matrix 128. |
| 5 | Arterial Spin Labelling with EPI readout | 5.5 | Labelling Slab 150mm, with a gap of 15mm and increasing delay times from 600 ms to 3050 ms - constant interval of 350 ms. Flip angle 40°; 3.5 × 3.5 × 6 mm3 voxels with a 1 mm gap between slices; 15 slices covering the cerebrum, TR 3500ms, TE 11ms, acq matrix 64×61, recon matrix 64. |
| 6 | GABA spectroscopy Frontal White Matter | 8.87 | Single Voxel MEGA-PRESS, voxel size 3x3x3cm3, TR 2000ms, TE 68ms, 44 blocks of 4 averages, interleaving decoupling and control MEGA pulses. |
| 7 | GABA spectroscopy Deep Grey Nuclei | 8.87 | As per 6 |

* includes scout sequences, planning and time for shim. GABA spectroscopy for sequence 6 and 7 related to differing voxel positioning.

***GABA Spectroscopy*** GABA was measured in two voxels of the brain; i) frontal white matter (FWM) and ii) deep grey nuclei (DGN) using the localized spectroscopy sequence MEGA-PRESS,[1] in which the dominant signal from creatine + phosphocreatine at 3.03 ppm, which overlaps the GABA C4 signal at 3.02 ppm, is suppressed by spectroscopic editing (Fig 1). The resultant edited spectrum consists of signals from glutamate and glutamine centred at 3.78 ppm (Glx), GABA at 3.02 ppm and an inverted signal from N-acetyl aspartate (NAA) at 2.02 ppm. For signal quantification, the AMARES algorithm implemented in jMRUI software[2] was utilized to calculate the concentrations of GABA+ (consisting mainly of GABA but with contributions from co-edited macro-molecule signals), the sum of glutamate plus glutamine (Glx) and NAA. The AMARES approach is a prior-knowledge based quantification method in the time domain, applied via nonlinear least square fitting. Regarding voxel localization, in order to maximize the signal to noise ratio in these studies we faced a necessary trade-off between the size of the voxel that could be interrogated against the time taken in the scanner to acquire the spectroscopic data. Our preliminary work when designing the study indicated that the best trade off was a 3cm x 3cm x 3cm voxel of interest which returned usable signal after an 8 minute acquisition. . Since we would not have been able to specifically acquire GABA data solely from the cortex given the lack of signal, we enlarged the interrogation voxel to that described above, and by necessity this would concentrate signal returen fromconcentrate the frontal white matter. Given previous studies have used similar voxel sizes [3, 4] and also concerntrated on the left frontal lobe in a similar region within the ASD population [3, 5, 6] we felt this voxel placement and size was appropriate[3, 4, 6].


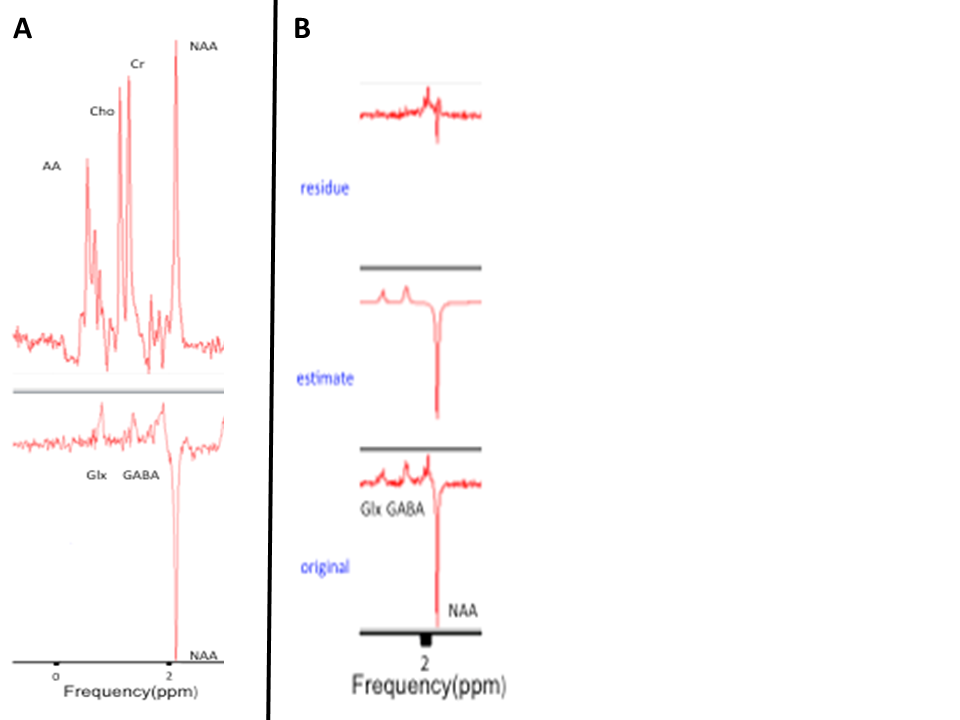

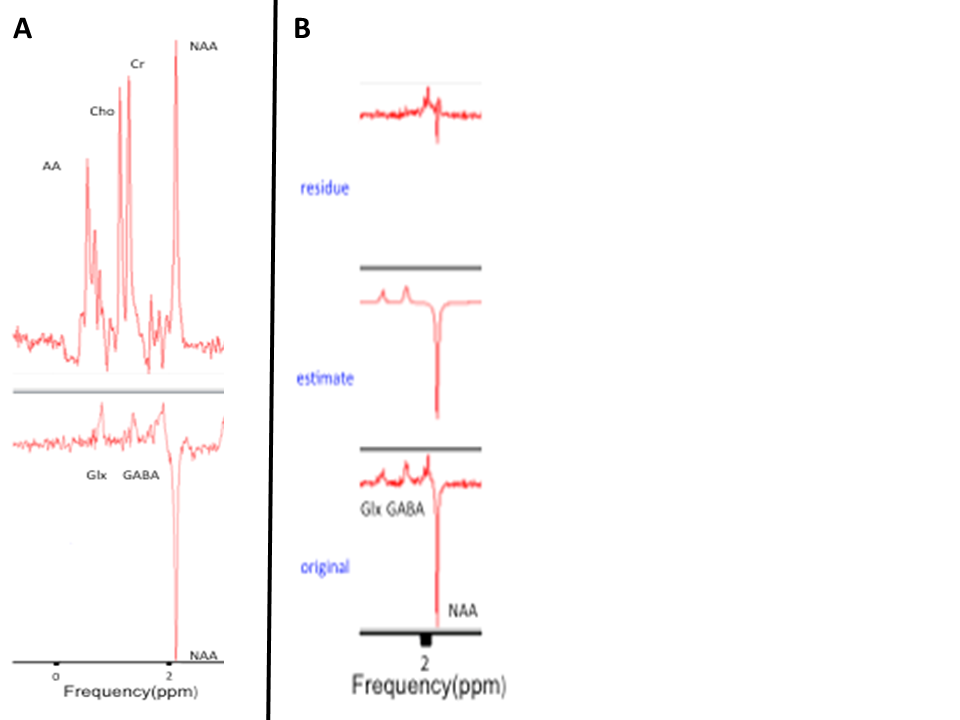


**Figure S1 a) *S****pectrum obtained from 3x3x3 voxel placed in deep gray matter of a 5 year old child using MEGA-PRESS suppression scheme at 3 T (top, non-edited sub-spectrum; bottom, GABA-edited spectrum) showing signals from amino-acid protons (AA), choline-containing compounds (cho), creatine + phosphocreatine (cr), N-acetylaspartate (NAA), GABA and glutamate + glutamine (glx).* **b)** *Figure depicting example output of AMARES Model fitting in jMRUI*

The semi-automatic post-processing steps for metabolite quantification within each VOI (Fig 1) included manual averaging of spectra, removal of water peak using the Hankel-Lanczos Singular Value Decomposition filter,[7] reference the NAA peak to 2.02 ppm, use of approximately 5 Hz apodization, and application of phase correction prior to jMRUI fitting. Prior knowledge on spectral parameters incorporated GABA+, which was modeled as a singlet with gaussian lineshape at 2.99 ppm, and glutamate plus glutamine (Glx), modeled as a doublet, with peaks at 3.71 and 3.78 ppm, and NAA set at 2 ppm as a single inverted Lorentzian peak. As contributions of macromolecules are expected when using MEGA-PRESS, the final quantification can only be described as GABA+ rather than GABA. A similar procedure to the aforementioned was undertaken to quantify NAA and other metabolite concentrations. The reference spectrum, used to convert from model-based to absolute concentration, was the signal from the non-suppressed water voxel. Participant datasets were excluded from analysis if there was severe extracranial lipid contamination, motion-related artefacts or if the post-fitting assessment indicated that standard deviation of the amplitude was greater than a threshold of 30%. Exclusion was also made if there was significant NF1 related T2 signal hyperintensity in the proposed region of interest. Finally, a procedure was followed in order to overlay each acquired MRS voxel onto the corresponding T1 weighted image so as to correct for cerebrospinal fluid (CSF) contamination. The partial volume segmentation software that was used was created by Dr. Nia Goulden and Dr. Paul Mullins of Bangor University (https://www.bangor.ac.uk/psychology/biu/Wiki.php.en). The software generates a GM, WM and CSF image and calculates the percentages of the aforementioned tissue types within each MRS voxel. Statistical analyses were performed with SPSS version 22.0 (SPSS Inc., Chicago, IL).


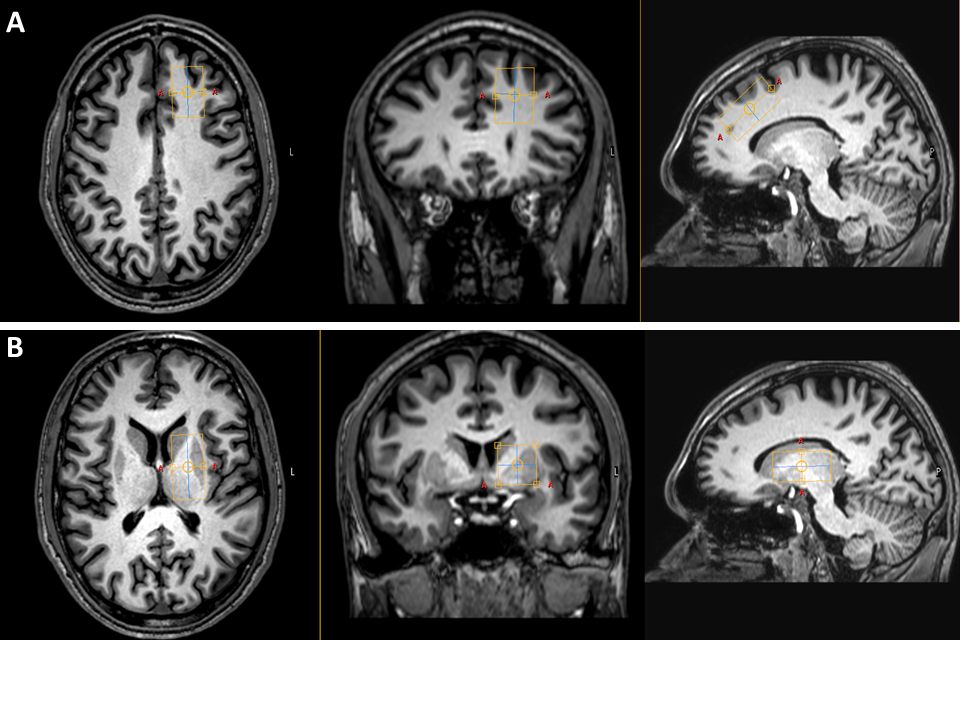


**Figure S2** *Example locations of VOI (3x3x3 cm^3^) acquired from a) left fontal white matter and b) deep grey matter (including caudate, lentiform nucleus, thalamus and putamen)*

We considered both the absolute change in values of the various metrics measured in

Table 1 from week zero to week twelve as well as the change in value of these features between the two time points, the null hypothesis being that if there was no effect of simvastatin on the brain then the distribution of changes should be the same between the two groups. This also allowed for the control of the wide variation in baseline measures. A student T-test was used for the absolute assessment of GABA values. With regards to the distribution of change two types of test were run. Firstly we ran an ANCOVA-type test, where an ordinary least-squares model of the form y = ax + bc was fit. Here x refers to the covariate of the initial value at week 0 and c refers to the group. Such a model makes normality and equal variance assumptions, therefore, we also applied the tests proposed by Vermeulen [8](a Mann-Whitney test with covariate adjustment) where the normality assumption is null.

***Resting state fMRI*** Table S1 shows the parameters of the multi-slice whole brain acquisition of these data. Spatial networks demonstrating strong temporal coactivation in the resting BOLD fMRI responses were defined using probabilistic independent component analysis (ICA). Data analysis was performed using the FSL software library[9] with MELODIC ICA decomposition.[10] Preprocessing included motion correction for each 4D time series, [11] brain extraction, spatial smoothing (gaussian full-width half maximum 4 mm), high pass temporal filtering, and registration to an age-specific paediatric template (Cincinnati Children’s Hospital, Imaging Research Center, irc.cchmc.org). Identification and removal of motion related independent components was performed with ICA-AROMA.[12] Temporal concatenation group ICA decomposed the pre-processed data into 25 independent components, performed separately on data acquired at week zero and week twelve (n=21, n=17 respectively). The default mode network (DMN) was identified by the characteristic pattern of coactivation involving medial prefrontal, posterior cingulate/precuneus, lateral parietal, lateral temporal, and cerebellar regions.[13, 14] For comparison, sensorimotor and medial visual networks were identified by characteristic coactivation patterns including perirolandic and medial occipital regions respectively.[14] The analysis for differences between groups was performed using a dual regression technique which allowed for voxel-by-voxel comparisons of functional connectivity.[15] Briefly, this involved using the spatial group ICA maps to derive subject specific temporal responses and thus estimate subject specific spatial maps. Component maps from different subjects could then be compared between groups using non-parametric permutation testing (5,000 permutations).

***Perfusion Imaging*** Perfusion imaging was achieved using arterial spin labelling (ASL). A pulsed STAR (signal targeting with alternating radiofrequency) labeling technique with Look-Locker readout[16] at eight timepoints was used for ASL-based perfusion imaging.[17] A series of excitation pulses were applied, with increasing delay times from 600 ms to 3050 ms, and a constant interval of 350 ms between them, followed by Look-Locker readout (TR/TE 350/11 ms,). To allow quantification of CBF a M0 (proton density) scan was acquired using a very similar multiphase ASL sequence but with TR of 10sec. The ASL analysis was carried out using in-house MATLAB (www.mathworks.com) routines. The raw images were qualitatively assessed for motion and artefacts before being processed with analysis using our previously published technique.[18] A single blood compartment model, was fit to the averaged images on a voxel-by-voxel basis, producing maps of Cerebral Blood Flow (CBF) and Arterial Arrival Time (AAT, without vascular crushing). Structural T1 images were processed using FreeSurfer v5.3.0 using a standardized and automated pipeline - recon all - of cortical surface-based analysis.[19] The output of this analysis generated forty five separate regions of interest which were used to guide the calculation of regional perfusion values using the 'Destrieux' cortical atlas [20] as per diffusion section.


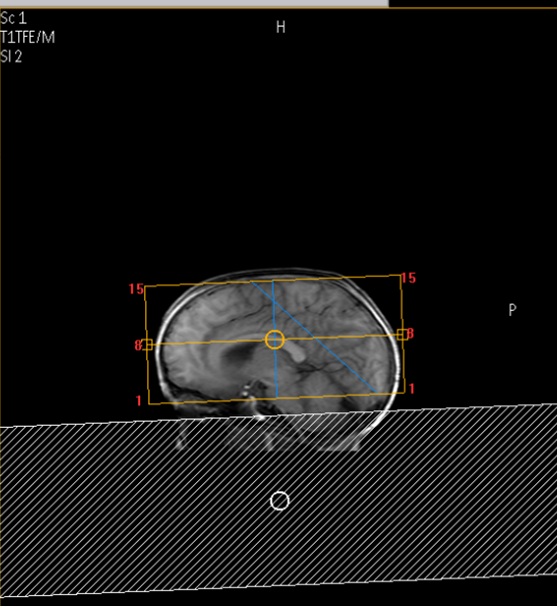


**Figure S3** *Example illustrating in sagittal view the position of the perfusion-imaging slices, which were planned above the ventricles and the labelling slab (150 mm) that was set 10 mm below the imaging slices*

***Diffusion Imaging*** The T1 images of each participant were preprocessed using the open source FreeSurfer v5.3.0 image analysis suite, which was downloaded and installed onto a Linux-based workstation. The image analysis suite encompasses a set of CT computational tools, which provide a standardized and automated pipeline - recon all - of cortical surface-based analysis.[21] The output of the analysis was used to guide the calculation of regional ADC values. FSL v.5 software was used for preprocessing the raw diffusion MR datasets. Eddy current correction was applied to the data, using the appropriate tool via the FSL software; [9] this tool is used to correct eddy current induced distortions and simple head motion by affine registration to the b0 image. The median ADC value of each of the fourty five areas was calculated via registration of ADC maps to freesurfer output; this was computed via FLIRT. [11]

***Judgement of Line Orientation (JLO)*** This test was used for assessment of visuospatial skills as reported in previous studies in NF1 cohorts. However, compliance with this test was low due to poor attentional skills and the younger age of the cohort. Due to these reasons and the amount of missing data, we do not report the results from this assessment.

**Table S2** *Baseline Descriptive Data*

|  | **Sample**  **N=30** | **Placebo**  **N=16** | **Simvastatin**  **N=14** |
| --- | --- | --- | --- |
| **Sex**  Male  Female | 24 (80%)  6 (20%) | 14 (87.5%)  2 (12.5%) | 10 (71.4%)  4 (28.6%) |
| **Mutation***  Inherited  De novo | 13 (43.3%)  16 (53.3%) | 10 (62.5%)  5 (31.3%) | 3 (21.4%)  11 (71.4%) |
| **Age** (years) | 8.10 (1.80) | 8.28 (1.76) | 7.90 (1.90) |
| **Weight** (kg) | 27.88 (7.59) | 29.85 (8.51) | 25.76 (6.08) |
| **Social Responsiveness Scale (SRS)** T Score (Mean, SD) | 83.00 (7.96) | 83.06 (7.58) | 82.93 (8.67) |
| **Autism Diagnostic Interview (ADI-R)** |  |  |  |
| Social Interaction (Total A) | 17.67 (5.16) | 18.88 (5.08) | 16.29 (5.08) |
| Social Communication (Total B) | 14.10 (4.14) | 14.19 (4.37) | 14.00 (4.02) |
| Restricted Repetitive Behaviours (Total C) | 5.90 (2.67) | 5.88 (2.78) | 5.93 (2.64) |
| **Autism Diagnostic Observation Schedule (ADOS)**  Social Affect  RRB  Total | 9.73(3.23)  2.00(1.67)  11.80(3.82) | 10.13(3.26)  1.88(1.63)  12.00(3.81) | 9.29 (3.24)  2.14(1.74)  11.57(3.96) |
| **WASI verbal IQ** (n=26) | 85.46 (12.72) | 81.57 (12.99) | 90.00 (11.24) |
| **Aberrant Behaviour Checklist (ABC) †** |  |  |  |
| Irritability | 21.72 (10.03) | 19.40 (10.38) | 24.21 (9.36) |
| Lethargy | 15.07 (7.62)^*^ | 14.20 (8.36) | 16.08 (6.85)^*^ |
| Stereotypy | 6.03 (4.44) | 4.87 (3.56) | 7.29 (5.04) |
| Hyperactivity | 27.03 (11.42) | 24.07 (13.04) | 30.21 (8.75) |
| Inappropriate speech | 6.66 (3.40) | 5.93 (3.15) | 7.43 (3.61) |
| **Clinical Global Impression (CGI)** Severity of Illness; mean (SD) | 3.73(0.785) | 3.88 (0.885) | 3.57(0.646) |
| **Parent-Defined Target symptoms** |  |  |  |
| Hyperactivity | 13 | 6 | 7 |
| Aggression | 13 | 6 | 7 |
| Social inappropriateness | 18 | 9 | 9 |
| Problems with communication | 5 | 3 | 2 |
| Inflexibility/obsessionality | 9 | 7 | 2 |
| Learning problems | 3 | 1 | 2 |
| **Conners 3 Parent Rating Scale†** |  |  |  |
| Inattention | 80.21 (10.70) | 79.73 (12.13) | 80.71 (9.36) |
| Hyperactivity | 76.34 (12.95) | 71.87 (14.75) | 81.14 (8.88) |
| **Riccardi Scale**  1  2  3  4 | 7 (23.3%) 17 (56.7%) 4 (13.3%) 2 6.7%) | 3 (18.8%) 12 (75%) 0 (0.0%) 1(6.3%) | 4 (28.6%) 5 (35.7%) 4 (28.6%) 1 (7.1%) |

Mutation status for 1 participant in placebo group unknown as participant was adopted

†1 observation missing for Aberrant Behaviour Checklist and Conners: N=29 overall, N=15 in Group A. ADOS, Autism Diagnostic Observation Scale. SRS T score >75 = autism symptoms within clinical range; ADOS total score >7 = meets diagnostic threshold for autism spectrum disorder.

High scores on WASI indicate better performance. High scores on SRS, ADI-R, ADOS, ABC and Conners are indicative of higher levels of impairment. Parent defined target symptoms- frequency of the problem as rated by the parent.

**Table S3:** *Baseline clinical findings*

|  | **Week 0 (N=30)** | **Placebo (N=16)** | **Simvastatin (N=14)** |
| --- | --- | --- | --- |
| **ABC†** |  |  |  |
| Irritability | 21.72 (10.03) | 19.40 (10.38) | 24.21 (9.36) |
| Lethargy | 15.07 (7.62)^*^ | 14.20 (8.36) | 16.08 (6.85)^*^ |
| Stereotypy | 6.03 (4.44) | 4.87 (3.56) | 7.29 (5.04) |
| Hyperactivity | 27.03 (11.42) | 24.07 (13.04) | 30.21 (8.75) |
| Inappropriate speech | 6.66 (3.40) | 5.93 (3.15) | 7.43 (3.61) |
|  |  |  |  |
| **Conners†** |  |  |  |
| Inattention | 80.21 (10.70) | 79.73 (12.13) | 80.71 (9.36) |
| Hyperactivity | 76.34 (12.95) | 71.87 (14.75) | 81.14 (8.88) |
| Learning problems | 72.66 (12.78) | 68.53 (10.78) | 77.07 (13.63) |
| Executive function | 76.66 (11.29) | 74.40 (12.15) | 79.07 (10.16) |
| Aggression | 72.17 (17.85) | 69.87 (17.92) | 74.64 (18.10) |
| Peer relations | 85.28 (9.62) | 83.53 (11.36) | 87.14 (7.27) |
|  |  |  |  |
| **CGI** |  |  |  |
| *Severity of illness (mean (SD)* | 3.73(0.785) | 3.88 (0.885) | 3.57(0.646) |

†1 observation missing for ABC and Conners: N=29 overall, N=15 in Group A
^*^1 additional observation missing

Higher scores on ABC, Conners & CGI are indicative of higher levels of impairment

**Table S4** *Adverse events*

| **Adverse events by body system** | **Simvastatin** | | **Placebo** | |
| --- | --- | --- | --- | --- |
|  | Grade 1-2 | Grade 3 | Grade 1-2 | Grade 3 |
| Gastrointestinal system disorders | 1 (1) | 0 | 4 (4) | 2 (2) |
| General, whole body system disorders | 1 (1) | 0 | 2 (2) | 0 |
| Neurologic system disorders | 6 (5) | 1 (1) | 12 (4) | 1 (1) |
| Musculoskeletal system disorders | 4 (2) | 0 | 4 (4) | 1 (1) |
| Respiratory system disorders | 2 (1) | 0 | 4 (4) | 2 (2) |
| Cardiovascular system disorders | 0 | 0 | 0 | 0 |
| Psychiatric disorders | 7 (7) | 2 | 13 (5) | 4 (3) |
| Visual system disorders | 0 | 0 | 0 | 0 |
| Dermatologic system disorders | 3 (3) | 2 (2) | 6 (4) | 1 (1) |
| Urinary system disorders | 0 | 0 | 1 (1) | 0 |
|  |  |  |  |  |

No of events (no of patients who reported the event)

**Table S5** *Week 4 intermediate outcomes*

| **Week 4 outcomes** | **Summary statistics** | | | **Mean difference** | | | |
| --- | --- | --- | --- | --- | --- | --- | --- |
|  | **Sample** | **Placebo** | **Simvastatin** | **Adjusted mean difference (95% CI)** | **Bootstrap SE** | **Effect Size (95% CI)** | **Number in analysis** |
| **ABC** | **N=29** | **N=16** | **N=13** |  |  |  |  |
| Irritability^*^ | 18.89 (10.02) | 17.06 (9.90) | 21.33(10.08)^*^ | 0.93 (-3.95, 5.82) | 2.49 | 0.09 (-0.39, 0.58) | 27 |
| Lethargy^*^ | 12.46 (8.95) | 11.56 (9.19) | 13.67 (8.86) ^*^ | 0.66 (-5.58, 6.91) | 3.19 | 0.07 (-0.62, 0.77) | 26 |
| Stereotypy^*^ | 6.00 (4.78) | 5.06 (4.36) | 7.25 (5.21) ^*^ | 0.42 (-2.21, 3.04) | 1.34 | 0.09 (-0.46, 0.64) | 27 |
| Hyperactivity | 24.41 (12.74) | 21.25 (12.82) | 28.31 (11.97) | 0.66 (-4.16, 5.48) | 2.46 | 0.05 (-0.33, 0.43) | 28 |
| Inappropriate speech | 5.69 (3.08) | 5.06 (2.82) | 6.46 (3.33) | 0.52 (-0.88, 1.92) | 0.71 | 0.17 (-0.29, 0.62) | 28 |
| 25% reduction irritability subscale | N=12 | 7 | 5 |  |  |  |  |
| **Conners** | **N=26** | **N=16** | **N=10** |  |  |  |  |
| Inattention | 76.88 (12.91) | 75.94 (12.97) | 78.40 (13.36) | 2.65 (-2.85, 8.16) | 2.81 | 0.21 (-0.22, 0.63) | 25 |
| Hyperactivity | 73.04 (16.00) | 70.31 (17.78) | 77.40 (12.22) | -2.67 (-9.02, 3.69) | 3.24 | -0.17 (-0.56, 0.23) | 25 |
| Learning problems | 68.38 (14.17) | 64.63 (11.19) | 74.40 (16.83) | 3.19 (-1.76, 8.15) | 2.53 | 0.23 (-0.12, 0.58) | 25 |
| Executive function | 74.19 (15.66) | 72.13 (17.55) | 77.50 (12.17) | -1.05 (-9.22, 7.12) | 4.17 | -0.07 (-0.59, 0.45) | 25 |
| Aggression | 71.04 (19.22) | 72.44 (19.67) | 68.80 (19.29) | -4.43 (-14.98, 6.11) | 5.38 | -0.23 (-0.78, 0.32) | 25 |
| Peer relations | 84.58 (11.06) | 83.44 (12.23) | 86.40 (9.19) | 1.10 (-3.15, 5.35) | 2.17 | 0.10 (-0.28, 0.48) | 25 |
| **Parent defined target symptoms (PDTS)** | **N=29** | **N= 16** | **N=13** |  |  |  |  |
| Mean (SD) | 4.38(1.179) | 4.46 (0.718) | 4.28 (1.577) |  |  |  |  |
| Responders (PDTS score <3) | 3 | 2 | 1 |  |  |  |  |
| **CGI**^*^ | **N=29** | **N=16** | **N=13** |  |  |  |  |
| *Severity of illness Mean (SD)* | 3.69(0.712) | 3.69 (0.793) | 3.69(0.630) |  |  |  |  |
| **Treatment responder** | N=1 | N=1 | N=0 |  |  |  |  |

^*^1 additional observation missing. Treatment responder defined as >25% reduction in ABC Irritability sub-scale and a score of improved or much improved on CGI. High scores on ABC, Conner’s, CGI & PDTS are indicative of higher levels of impairment.

**Table S6:** *Quantification of MAPK outcomes at baseline and endpoint*

| **Participant number** | **Group** | **MAPKKinase_Wk_0** | **MAPKinase_**  **Wk_12** |
| --- | --- | --- | --- |
| S0001 | Placebo | 50.3114126 | .2188209 |
| S0002 | Placebo | 2.9527485 | 4.8079458 |
| S0006 | Placebo | 3.8926110 | 61.8679608 |
| S0020 | Placebo | 27.5060898 | 142.9943580 |
| S0023 | Placebo | 3.0623875 | 42.2400651 |
| S0029 | Placebo | 63.7154251 | 70.4316268 |
| S0033 | Placebo | 96.9558036 |  |
| S0034 | Placebo | 35.0355789 | 92.9567297 |
| S0035 | Placebo | 56.1097401 | 64.3669527 |
| S0036 | Placebo | 45.2664300 | 89.7891294 |
| S0037 | Placebo | 62.6400898 |  |
| S0046 | Placebo | 10.9267266 | 48.8793436 |
| S0056 | Placebo | 15.7506385 | 31.6528862 |
| S0057 | Placebo | 119.4835133 | 376.8717635 |
| S0060 | Placebo | 167.6537279 | 56.7577496 |
| S0063 | Placebo |  |  |
| S0005 | Simvastatin | 4.5859668 | 12.1169060 |
| S0010 | Simvastatin | 24.1460914 | 9.0388298 |
| S0011 | Simvastatin | 2.2530488 | 18.8252183 |
| S0021 | Simvastatin |  |  |
| S0025 | Simvastatin | 4.5638149 | 85.4512618 |
| S0027 | Simvastatin | 54.9854703 |  |
| S0031 | Simvastatin | 25.6439249 | 31.2619203 |
| S0038 | Simvastatin | 62.7051706 | 61.4602927 |
| S0039 | Simvastatin | 51.8592134 |  |
| S0043 | Simvastatin | 355.4792373 | 47.5177311 |
| S0051 | Simvastatin |  |  |
| S0058 | Simvastatin | 172.9821432 | 75.6083354 |
| S0061 | Simvastatin | 47.9587414 | 72.1282853 |
| S0062 | Simvastatin | 86.8384461 |  |

**Table S7:** *A comparison of the mutation data in the SANTA sample to previously reported data from a clinic referred NF1 sample (see text)*

| **Study Ref** | **Mutation** | **Type** | **Exon** |
| --- | --- | --- | --- |
| S0001 | WGD | large re | EGD |
| S0003 | c.3911T>A p. (Leu 1 304Ter) | nonsense | exon 29 |
| S0005 | c.2041C>T | nonsense | exon 18 |
| S0006* | c.3741deIT | frameshift | exon 28 |
| S0010 | c.3916C>T | nonsense | exon 29 |
| S0011 | c.5749+332A>G | splice | intron 38 |
| S0020 | c.3497-4T>G | splice | intron 26 |
| S0021 | c.203delins6 | frameshift | exon 02 |
| S0023 | c.2504_2505delAG | frameshift | exon 21 |
| S0025 | c.2953C>T p.(Gln985Ter) | nonsense | exon 22 |
| S0027 | c.174delC | frameshift | exon 02 |
| S0029** | C.7908-321C>G | Splice deep intronic | intron 54 |
| S0031 | c.5546G>A p.(Arg1849G1n) | missense | exon 37 |
| S0033 | c.4537C>T(p.Arg1513Ter) | nonsense | exon 35 |
| S0034 | c.1246C>Tp.(Arg416Ter) | nonsense | exon 11 |
| S0035* | c.3741delT | frameshift | exon 28 |
| S0036 | c.7239_7240insTT | frameshift | exon 49 |
| S0037 | c.1381C>Tp.(Arg461Te | nonsense | exon 12 |
| S0038 | c.2087G>A p.(Trp696Ter) | nonsense | exon 18 |
| S0039 | c.4306A>G p.(Lys1436GIu) | missense | exon 32 |
| S0043 | c.1260+1G>A | splice | intron 11 |
| S0046** | C.7908-321C>G | Splice deep intronic | intron 54 |
| S0051 | Not done |  |  |
| S0056 | c.479G>Tp.(Arg160Met) | missense | intron 4 |
| S0057 | C7404_7405insTA. Glu2469Ter | frameshift | Exon 51 |
| S0058 | c.7096_7101del6p.(Asn2366_Phe2367del) | in-frame deletion | exon 48 |
| S0060 | c.1756_1759delACTA | frameshift | exon 16 |
| S0061 | c.1186-1G>T | splice | Exon 11 |
| S0062 | Not done |  |  |
| S0063 | Not done |  |  |

Data was not available for 3 participants

* & ** sibling pairs

|  | **SANTA** | **%** | **Controls+** | **%** |
| --- | --- | --- | --- | --- |
| splice | 5 | 19.23% | 80 | 22.99% |
| frameshift | 8 | 30.77% | 114 | 32.76% |
| nonsense | 8 | 30.77% | 76 | 21.84% |
| missense | 3 | 11.54% | 31 | 8.91% |
| large re | 1 | 3.85% | 28 | 8% |
| ifd | 1 | 3.85% | 13 | 3.74% |
|  | 26 |  | 342 |  |

 + Evans et al European Journal of Human Genetics 2016 [22]

**Figure S4** *SANTA CONSORT flow diagram (see text; SRS, Social Responsiveness Scale)*

**Ineligible on SRS (SRS T scores<59) N= 11**

**Older than 10.5 years N=4**

**On active exclusion treatment (see text) N=3**

**Not NF1 n=2**

**Screened on SRS**

**N= 91**

**Met research ASD criteria N=34**

**Did not meet research ASD criteria N=19**

**In-depth ASD assessments**

**N=53**

**Met eligibility criteria N= 71**

**Consent withdrawn after baseline visit N=1**

**DNA week 12 assessment N=2**

**Consent withdrawn before randomisation N=2**

**Not NF1 diagnosis N=2**

**DNA in-depth assessment n=7**

**Not interested N=11**

**Placebo**

**N=16**

**DNA week 12 assessment N=1**

**Simvastatin**

**N= 14**

**Completed 3 trial visits N=15**

**Completed 3 trial visits**

**N=11**

**References**

1. Mullins PG, McGonigle DJ, O'Gorman RL, Puts NA, Vidyasagar R, Evans CJ, et al. Current practice in the use of MEGA-PRESS spectroscopy for the detection of GABA. NeuroImage. 2014;86:43-52.

2. Naressi A, Couturier C, Devos JM, Janssen M, Mangeat C, de Beer R, et al. Java-based graphical user interface for the MRUI quantitation package. Magma. 2001;12(2-3):141-52.

3. Harada M, Taki MM, Nose A, Kubo H, Mori K, Nishitani H, et al. Non-invasive evaluation of the GABAergic/glutamatergic system in autistic patients observed by MEGA-editing proton MR spectroscopy using a clinical 3 tesla instrument. J Autism Dev Disord. 2011;41(4):447-54.

4. Gaetz W, Bloy L, Wang DJ, Port RG, Blaskey L, Levy SE, et al. GABA estimation in the brains of children on the autism spectrum: measurement precision and regional cortical variation. NeuroImage. 2014;86:1-9.

5. Hassan TH, Abdelrahman HM, Fattah NRA, El-Masry NM, Hashim HM, El-Gerby KM, et al. Blood and brain glutamate levels in children with autistic disorder. Research in autism spectrum disorders. 2013;7(4):541-8.

6. Fatemi SH, Reutiman TJ, Folsom TD, Thuras PD. GABA(A) receptor downregulation in brains of subjects with autism. J Autism Dev Disord. 2009;39(2):223-30.

7. Pijnappel WWF, Vandenboogaart A, Debeer R, Vanormondt D. Svd-Based Quantification of Magnetic-Resonance Signals. J Magn Reson. 1992;97(1):122-34.

8. Vermeulen K, Thas O, Vansteelandt S. Increasing the power of the Mann-Whitney test in randomized experiments through flexible covariate adjustment. Statistics in medicine. 2015;34(6):1012-30.

9. Smith SM, Jenkinson M, Woolrich MW, Beckmann CF, Behrens TE, Johansen-Berg H, et al. Advances in functional and structural MR image analysis and implementation as FSL. NeuroImage. 2004;23 Suppl 1:S208-19.

10. Beckmann CF, Smith SM. Probabilistic independent component analysis for functional magnetic resonance imaging. IEEE transactions on medical imaging. 2004;23(2):137-52.

11. Jenkinson M, Smith S. A global optimisation method for robust affine registration of brain images. Medical image analysis. 2001;5(2):143-56.

12. Pruim RH, Mennes M, Buitelaar JK, Beckmann CF. Evaluation of ICA-AROMA and alternative strategies for motion artifact removal in resting state fMRI. NeuroImage. 2015;112:278-87.

13. Raichle ME, MacLeod AM, Snyder AZ, Powers WJ, Gusnard DA, Shulman GL. A default mode of brain function. Proceedings of the National Academy of Sciences of the United States of America. 2001;98(2):676-82.

14. Smith SM, Fox PT, Miller KL, Glahn DC, Fox PM, Mackay CE, et al. Correspondence of the brain's functional architecture during activation and rest. Proceedings of the National Academy of Sciences of the United States of America. 2009;106(31):13040-5.

15. Beckmann CF, Mackay CE, Filippini N, Smith S. Group comparison of resting-state fMRI data using multi-subject ICA and dual regression. NeuroImage. 2009;47(S148).

16. Look DC, Locker DR. Time saving in measurement of NMR and EPR relaxation times. Review of Scientific Instruments. 1970;41(250).

17. Petersen ET, Lim T, Golay X. Model-free arterial spin labeling quantification approach for perfusion MRI. Magnetic resonance in medicine. 2006;55(2):219-32.

18. Vidyasagar R, Abernethy L, Pizer B, Avula S, Parkes LM. Quantitative measurement of blood flow in paediatric brain tumours-a comparative study of dynamic susceptibility contrast and multi time-point arterial spin labelled MRI. The British journal of radiology. 2016;89(1062):20150624.

19. Dale AM, Fischl B, Sereno MI. Cortical surface-based analysis. I. Segmentation and surface reconstruction. NeuroImage. 1999;9(2):179-94.

20. Fischl B, van der Kouwe A, Destrieux C, Halgren E, Segonne F, Salat DH, et al. Automatically parcellating the human cerebral cortex. Cerebral cortex. 2004;14(1):11-22.

21. Fischl B, Sereno MI, Dale AM. Cortical surface-based analysis. II: Inflation, flattening, and a surface-based coordinate system. NeuroImage. 1999;9(2):195-207.

22. Evans DG, Bowers N, Burkitt-Wright E, Miles E, Garg S, Scott-Kitching V, et al. Comprehensive RNA Analysis of the NF1 Gene in Classically Affected NF1 Affected Individuals Meeting NIH Criteria has High Sensitivity and Mutation Negative Testing is Reassuring in Isolated Cases With Pigmentary Features Only. EBioMedicine. 2016;7:212-20.
